# Genetic variants in migraine: a field synopsis and systematic re-analysis of meta-analyses

**DOI:** 10.1186/s10194-020-01087-5

**Published:** 2020-02-11

**Authors:** Yating Zhao, Ruixia Zhu, Tongling Xiao, Xu Liu

**Affiliations:** Department of Neurology, First Affiliated Hospital of China Medical University, No. 155 North Nanjing Street, Shenyang, 110001 Liaoning China

**Keywords:** Genetic variant, Migraine, Meta-analysis, FPRP, BFDP, GWAS

## Abstract

**Objective:**

Numerous genetic variants from meta-analyses of observational studies and GWAS were reported to be associated with migraine susceptibility. However, due to the random errors in meta-analyses, the noteworthiness of the results showing statistically significant remains doubtful. Thus, we performed this field synopsis and re-analysis study to evaluate the noteworthiness using a Bayesian approach in hope of finding true associations.

**Methods:**

Relevant meta-analyses from observational studies and GWAS examining correlation between all genetic variants and migraine risk were included in our study by a PubMed search. Identification of noteworthy associations were analyzed by false-positive rate probability (FPRP) and Bayesian false discovery probability (BFDP). Using noteworthy variants, GO enrichment analysis were conducted through DAVID online tool. Then, the PPI network and hub genes were performed using STRING database and CytoHubba software.

**Results:**

As for 8 significant genetic variants from observational studies, none of which showed noteworthy at prior probability of 0.001. Out of 47 significant genetic variants in GWAS, 36 were noteworthy at prior probability of 0.000001 via FPRP or BFDP. We further found the pathways “positive regulation of cytosolic calcium ion concentration” and “inositol phosphate-mediated signaling” and hub genes including *MEF2D*, *TSPAN2*, *PHACTR1*, *TRPM8* and *PRDM16* related to migraine susceptibility.

**Conclusion:**

Herein, we have identified several noteworthy variants for migraine susceptibility in this field synopsis. We hope these data would help identify novel genetic biomarkers and potential therapeutic target for migraine.

## Introduction

Migraine is a complex and incapacitating neurologic condition with more than one billion individuals suffering from and imposes a huge socioeconomic burden worldwide [1, 2]. It is characterized by recurrent episodes of unilateral throbbing pain often accompanied with nausea, phonophobia and photophobia, leading to a decline in life quality or even disability [3]. In the Global Burden of Disease (GBD) 2015, migraine ranked the seventh among the leading causes of years lived with disability for all ages and the third for ages 15 to 49 years [4]. Recently, it has been reported that, in the United States, migraine affected almost 15% adults and the annual economic cost was over $ 2600 each person diagnosed with episodic migraine and $ 8000 for those with chronic migraine [5, 6]. Thus, a large amount of studies was performed to explore the risk factors and pathogenesis of migraine over the past decades.

Except for the common risk factors such as obesity, medication overuse, poor sleep, caffeine and stressful life events, the genetic factors for migraine susceptibility was drawing more and more attention [7, 8]. Numerous genetic polymorphisms from meta-analyses of observational studies and GWAS were reported to be associated with migraine susceptibility [9]. However, due to the random errors in meta-analyses leading to false-positive results, the noteworthiness of the results with statistical significance remains doubtful. Therefore, in this field synopsis, we summarized and re-analyzed all significant genetic variants from meta-analyses of observational studies and GWAS, then assessed their noteworthiness using Bayesian procedures including false-positive rate probability (FPRP) and Bayesian false discovery probability (BFDP) and discussed possible molecular mechanisms for migraine occurrence.

## Methods

### Search strategy and data extraction

A comprehensive literature search was conducted in the PubMed database up to 31 July 2019, by using the following terms: “migraine/ headache” and “meta-analysis” and “polymorphism/genome-wide association study/SNP/GWAS/variant/allele/genotype”. Studies were all selected according to the following criteria: (1) meta-analysis design study; (2) evaluating the association between genetic polymorphisms and migraine risk; (3) raw data available including odds ratios (ORs), 95% confidence intervals (CIs) or other information necessary for FPRP and BFDP calculation; (4) studies published in English. Data including author, published year, genetic variant, genetic model, OR, 95% CI, *P*-value, ethnicity, type of migraine, number of cases and controls, heterogeneity and publication bias were extracted from the meta-analyses included according to above criteria.

### Assessment methods for meta-analysis

In order to figure out the noteworthiness of meta-analysis on the association between genetic polymorphisms and migraine susceptibility, two novel statistic methods, FPRP and BFDP, were applied in our study. FPRP is the probability that no true association exists between genetic variant and disease drawing a statistically significant finding [10]. The magnitude of the FPRP is determined by prior probability, statistical power and observed *P*-value. Owing to the highly subjective prior probability, we analyzed a wide range of prior probability. We calculated FPRP values using two levels of prior probabilities: at 0.05/0.001 (medium/low prior level) that would be expected for candidate SNPs, and at 0.001/0.000001 (medium/low prior level) for GWAS SNPs. Moreover, the lower the prior probability, the more reliable the result. Besides, we used statistical power to detect ORs of 1.2 and 1.5 for computing FPRP at each prior probability. For the statistically significant SNPs (95% CI that excluded 1 and *P*-value which was lower than 5 × 10^− 8^ for meta-analysis of GWAS or 0.05 for observational studies), we calculated FPRP by using the Excel spreadsheet offered by Wacholder (http://jncicancerspectum.oupjournals.org/jnci/content/vol96/issue6) [10]. FPRP values lower than 0.2 were considered to be noteworthy.

BFDP is the fact that if an association is reported as noteworthy, BFDP is the probability of a false discovery [11]. Relevant data and prior probability applied in the calculation of BFDP were same as it of FPRP. The BFDP was estimated by using the excel Calculation Spreadsheet (http://faculty.washington.edu/jonno/cv.html) [11]. And BFDP with the values of less than 0.8 were considered to be a noteworthy significant association. Different from FPRP, BFDP is a new statistical method based on logistic regression model rather than standard normal distribution and doesn’t rely on the statistical power. Thus, BFDP has a sounder methodological basis. Nevertheless, Wakefield admits that there is no significant difference in the overall behavior between these two approaches [11]. Therefore, we presented the both results of FPRP and BFDP which allowed readers to reach a more comprehensive judgment.

Besides, for the meta-analysis of observational studies, summary evidence was also evaluated using Venice criteria, which have been described in detail previously [12, 13]. Briefly, we classified the strength of credibility into A, B and C grades that were separately characterized as strong, moderated and weak in three parameters including amount of evidence, replication of association and the protection from bias. According to this criteria, high credibility was defined as including A grades only, intermediate credibility was composed of A and B grades and low credibility was one or more C grades.

### Joint population attributable risk calculation

We evaluated the cumulative effect of all noteworthy SNPs on migraine susceptibility. We used the minor allelic frequency (MAF) to calculated the population attributable risk (PAR) and further estimated the Joint PAR% for the SNPs showing noteworthiness during the computing of BFDP (BFDP < 0.8) or FPRP (FPRP< 0.2) at a prior probability of 10^− 6^ assumed for GWAS SNPs and 10^− 3^ for candidate SNPs at a statistical power to detect the OR of 1.5.

### GO and enrichment pathway analysis and PPI network construction

Gene ontology (GO) analysis is a useful bioinformatic method for annotating genes. In our study, GO enrichment analyses were carried out using a list of genes with noteworthy SNPs through DAVID online tool (http://david.abcc.ncifcrf.gov/). Then, we applied the STRING 11.0 network database to construct a protein-protein interaction (PPI) network. We set the minimum required interaction score at 0.15, no more than five interactors and four active interaction sources (experiments, text mining, co-expression and database) for PPI construction. Finally, we used the Cytoscape (version 3.4.2) and cytoHubba to detect hub genes meanwhile the cut-off criterion of hub genes was setting as degree ≥7.

## Results

**Fig. 1:**
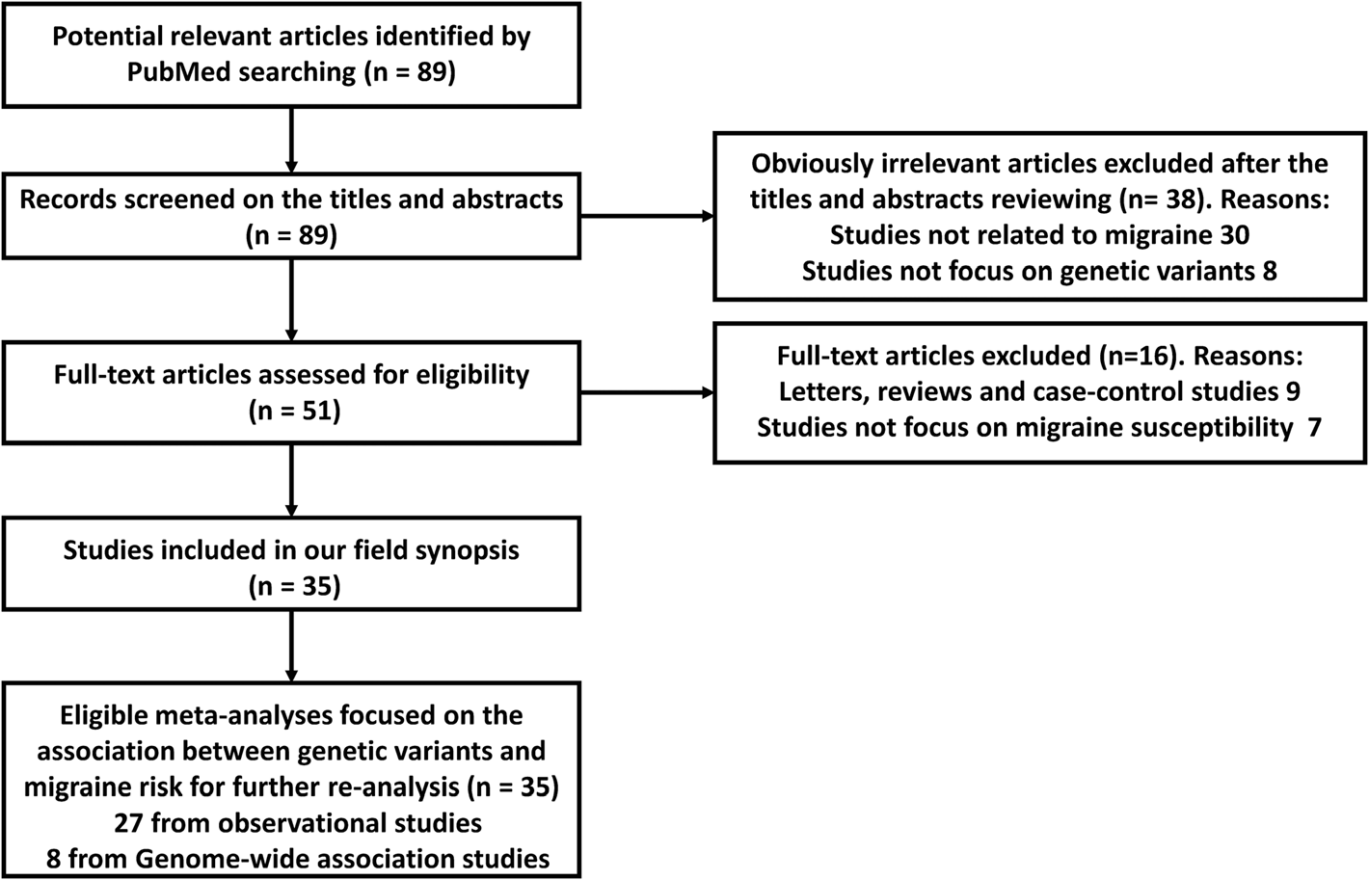
The flow chart of the articles screening and selection

First, a total of 89 articles were identified according to our search strategy. Second, 38 obviously irrelevant articles were excluded by screening the titles and abstracts. After reviewing the full-text articles, 16 were further excluded, among which 9 articles were not meta-analyses and 7 were not correlated with migraine susceptibility. At last, 35 articles were selected into our study [14–48]. The screening process of the articles was performed by two independent researchers and shown in Fig. 1. Overall data retrieved from meta-analyses of observational studies and GWAS on risk of migraine were summarized in Additional file 1: Table S1 and Additional file 2: Table S2. Third, when more than one meta-analysis was available for certain SNP, we only included the results from more recent meta-analysis with larger sample size. Finally, the statistically significant results with *P* < 0.05 for observational studies and *P* < 5 × 10^− 8^ for GWAS were summarized in Tables 1 and 2.

**Table 1:** Meta-analyses results of genetic variants with statistically significance (*P*-value< 0.05) from observational studies

Author, year	Gene/variant	Comparison	OR (95%CI)	*P*-Value	Ethnicity	No. of cases/controls	FPRP Values at Prior probability				BFDP 0.05	BFDP 0.001	Venice criteria	Venice criteria score
							OR 1.2		OR 1.5					
							0.05	0.001	0.05	0.001				
Liu L, 2019 [48]	*MTHFR*/rs1801133	T vs. C	1.19 (1.06–1.33)	0.004	Overall 26 (Caucasian 20, Asian 6)	10,228/28608	0.069	0.795	0.040	0.685	0.476	0.980	A + C + C	Low
Liu L, 2019 [48]	*MTHFR*/rs1801133	TT vs. CT + CC	1.29 (1.06–1.56)	0.010	Overall 26 (Caucasian 20, Asian 6)	10,228/28608	0.419	0.974	0.149	0.902	0.726	0.993	A + C + C	Low
Liu L, 2019 [48]	*MTHFR*/rs1801133	TT + CT vs. CC	1.17 (1.02–1.35)	0.027	Overall 26 (Caucasian 20, Asian 6)	10,228/28608	0.485	0.980	0.375	0.969	0.880	0.997	A + C + C	Low
Liu L, 2019 [48]	*MTHFR*/rs1801133	TT vs. CC	1.32 (1.07–1.64)	0.011	Overall 26 (Caucasian 20, Asian 6)	10,228/28608	0.543	0.984	0.209	0.933	0.778	0.995	A + C + C	Low
Liu L, 2019 [48]	*MTHFR*/rs1801131	CC vs. AC + AA	1.82 (1.09–3.04)	0.022	Overall 5 (Caucasian 4, Asian 1)	1368/1411	0.883	0.998	0.647	0.990	0.899	0.998	A + C + C	Low
Liu L, 2019 [48]	*MTHFR*/rs1801131	CC vs. AA	1.78 (1.03–3.07)	0.038	Overall 5 (Caucasian 4, Asian 1)	1368/1411	0.903	0.998	0.729	0.993	0.917	0.998	A + C + C	Low
Gao X, 2018 [47]	*GRIA1*/rs2195450	CT vs. CC	1.23 (1.02–1.48)	0.03	Overall 4 (Caucasian 3, Asian 1)	963/1167	0.576	0.986	0.354	0.966	0.862	0.997	A + B + A	Intermediate
Terrazzino S, 2017 [44]	*BDNF*/rs6265	A vs. G	1.17 (1.03–1.34)	0.014	Overall 5 (Caucasian 5)	2884/3760	0.408	0.973	0.307	0.959	0.856	0.997	A + A + A	High
Terrazzino S, 2017 [44]	*BDNF*/rs6265	AA + GA vs. GG	1.22 (1.05–1.41)	0.011	Overall 5 (Caucasian 5)	2884/3760	0.247	0.945	0.119	0.877	0.694	0.992	A + A + A	High
Cai X, 2017 [42]	*BDNF*/rs2049046	A vs. T	0.88 (0.79–0.98)	0.02	Overall 4 (Caucasian 4)	1260/1380	0.311	0.960	0.275	0.952	0.855	0.997	A + A + C	Low
Cai X, 2017 [42]	*BDNF*/rs2049046	AA vs. TA + TT	0.80 (0.67–0.96)	0.02	Overall 4 (Caucasian 4)	1260/1380	0.486	0.980	0.243	0.944	0.809	0.996	A + A + A	High
Cai X, 2017 [42]	*BDNF*/rs2049046	AA vs. TT	0.78 (0.62–0.97)	0.02	Overall 4 (Caucasian 4)	1260/1380	0.637	0.989	0.345	0.965	0.853	0.997	A + A + A	High
Cai X, 2017 [42]	*BDNF*/rs2049046	AA vs. TA	0.81 (0.67–0.99)	0.03	Overall 4 (Caucasian 4)	1260/1380	0.658	0.990	0.436	0.976	0.887	0.998	A + A + A	High
Li L, 2015 [37]	*ESR1*/rs1801132	GG vs. CC	1.51 (1.15–1.99)	0.003	Overall 5 (Caucasian 4, Asian 1)	2027/1919	0.559	0.985	0.119	0.877	0.644	0.990	A + A + A	High
Li L, 2015 [37]	*ESR1*/rs1801132	GG vs. CG + CC	1.52 (1.16–1.98)	0.002	Overall 5 (Caucasian 4, Asian 1)	2027/1919	0.477	0.980	0.073	0.805	0.542	0.984	A + B + A	Intermediate
Li L, 2015 [37]	*ESR1*/rs2228480	AG vs. GG	1.14 (1.01–1.28)	0.030	Overall 6 (Caucasian 5, Asian 1)	2293/2026	0.385	0.971	0.336	0.964	0.877	0.997	A + A + A	High
Li L, 2015 [37]	*ESR1*/rs2228480	AA + AG vs. GG	1.13 (1.00–1.26)	0.003	Overall 6 (Caucasian 5, Asian 1)	2293/2026	0.381	0.970	0.346	0.965	0.885	0.998	A + B + A	Intermediate
Liu H, 2011 [23]	*5-HTT*/VNTR	Stin2.12 allele	1.34 (1.09–1.64)	0.006	Overall 4 (Caucasian 3, Asian1)	495/729	0.377	0.970	0.090	0.840	0.628	0.989	A + A + NA	NA
Liu H, 2011 [23]	*5-HTT*/VNTR	12/12 genotype	1.55 (1.17–2.05)	0.002	Overall 4 (Caucasian 3, Asian1)	495/729	0.526	0.983	0.090	0.838	0.557	0.986	A + A + NA	NA

*OR* odds radio, *CI* confidence interval, *FPRP* false-positive rate probability, *BFDP* Bayesian false discovery probability, *NA* not available

**Table 2:** Meta-analyses results of genetic variants with statistically significance (*P*-value< 5*10^−8^) from GWAS

Author, year	Gene/variant	Comparison	OR (95%CI)	*P*-Value	Ethnicity	No. of cases/controls	FPRP Values at Prior probability				BFDP 0.001	BFDP 0.000001
							OR 1.2		OR 1.5			
							0.001	0.000001	0.001	0.000001		
Chang X, 2018 [45]	*NMUR2*/rs1946225	G vs. T	2.29 (1.73–3.05)	9.55E-09	Overall 3 (European 1, African 2)	1212/13494	0.717	1.000	0.006	0.851	0.027	0.965
Chang X, 2018 [45]	*NMUR2*/rs72793414	A vs. G	2.44 (1.85–3.23)	3.81E-10	Overall 3 (European 1, African 2)	1212/13494	0.545	0.999	0.001	0.541	0.003	0.743
Gormley P, 2016 [40]	*LRP1*/rs11172113	C vs. T	0.90 (0.89–0.91)	5.6E-49	Overall (Caucasian 22)	59,674/316078	–	–	–	–	6.004E-72	6.011E-69
Gormley P, 2016 [40]	*PRDM16*/rs10218452	G vs. A	1.11 (1.10–1.13)	5.3E-38	Overall (Caucasian 22)	59,674/316078	–	–	–	–	8.348E-25	8.356E-22
Gormley P, 2016 [40]	*FHL5*/rs67338227	T vs. A	1.09 (1.08–1.11)	2.0E-27	Overall (Caucasian 22)	59,674/316078	–	–	–	–	4.430E-15	4.435E-12
Gormley P, 2016 [40]	*TSPAN2*/rs2078371	C vs. T	1.11 (1.09–1.13)	4.1E-24	Overall (Caucasian 22)	59,674/316078	–	–	–	–	8.348E-25	8.356E-22
Gormley P, 2016 [40]	*TRPM8*/rs10166942	C vs. T	0.94 (0.89–0.99)	1.0E-23	Overall (Caucasian 22)	59,674/316078	0.951	1.000	0.951	1.000	0.998	1.000
Gormley P, 2016 [40]	*PHACTR1*/rs9349379	G vs. A	0.93 (0.92–0.95)	5.8E-22	Overall (Caucasian 22)	59,674/316078	2.308E-08	2.310E-05	2.308E-08	2.310E-05	4.007E-06	3.995E-03
Gormley P, 2016 [40]	*MEF2D*/rs1925950	A vs. G	1.07 (1.06–1.09)	9.1E-22	Overall (Caucasian 22)	59,674/316078	8.014E-10	8.022E-07	8.014E-10	8.022E-07	1.694E-07	1.695E-04
Gormley P, 2016 [40]	*SLC24A3*/rs4814864	C vs. G	1.07 (1.06–1.09)	2.2E-19	Overall (Caucasian 22)	59,674/316078	8.014E-10	8.022E-07	8.014E-10	8.022E-07	1.694E-07	1.695E-04
Gormley P, 2016 [40]	*FGF6*/rs1024905	A vs. G	1.06 (1.04–1.08)	2.1E-17	Overall (Caucasian 22)	59,674/316078	9.959E-07	9.959E-04	9.959E-07	9.959E-04	1.767E-04	0.150
Gormley P, 2016 [40]	*C7orf10*/rs186166891	T vs. A	1.09 (1.07–1.12)	9.7E-16	Overall (Caucasian 22)	59,674/316078	4.993E-07	4.935E-04	4.993E-07	4.935E-04	6.430E-05	6.048E-02
Gormley P, 2016 [40]	*PLCE1*/rs10786156	G vs. C	0.95 (0.94–0.96)	2.0E-14	Overall (Caucasian 22)	59,674/316078	–	–	–	–	3.842E-16	3.846E-13
Gormley P, 2016 [40]	*KCNK5*/rs10456100	T vs. C	1.06 (1.04–1.07)	6.9E-13	Overall (Caucasian 22)	59,674/316078	–	–	–	–	3.372E-28	3.376E-25
Gormley P, 2016 [40]	*ASTN2*/rs6478241	T vs. A	1.05 (1.04–1.07)	1.2E-12	Overall (Caucasian 22)	59,674/316078	4.011E-04	0.287	4.011E-04	0.287	0.055	0.983
Gormley P, 2016 [40]	*MRVI1*/rs4910165	G vs. C	0.94 (0.91–0.98)	2.9E-11	Overall (Caucasian 22)	59,674/316078	0.783	1.000	0.783	1.000	0.993	1.000
Gormley P, 2016 [40]	*HPSE2*/rs12260159	A vs. G	0.92 (0.89–0.94)	3.2E-10	Overall (Caucasian 22)	59,674/316078	2.979E-11	2.982E-08	2.979E-11	2.982E-08	5.906E-09	5.912E-06
Gormley P, 2016 [40]	*CFDP1*/rs77505915	A vs. T	1.05 (1.03–1.06)	3.3E-10	Overall (Caucasian 22)	59,674/316078	–	–	–	–	3.476E-18	3.479E-15
Gormley P, 2016 [40]	*RNF213*/rs17857135	C vs. T	1.06 (1.04–1.08)	5.2E-10	Overall (Caucasian 22)	59,674/316078	9.959E-07	9.959E-04	9.959E-07	9.959E-04	1.767E-04	0.150
Gormley P, 2016 [40]	*NRP1*/rs2506142	G vs. A	1.06 (1.04–1.07)	1.5E-09	Overall (Caucasian 22)	59,674/316078	–	–	–	–	3.372E-28	3.376E-25
Gormley P, 2016 [40]	*GPR149*/rs13078967	C vs. A	0.87 (0.83–0.91)	1.8E-09	Overall (Caucasian 22)	59,674/316078	1.300E-06	1.299E-03	1.260E-06	1.260E-03	1.117E-04	0.101
Gormley P, 2016 [40]	*JAG1*/rs111404218	G vs. C	1.05 (1.03–1.07)	2.0E-09	Overall (Caucasian 22)	59,674/316078	4.011E-04	0.287	4.011E-04	0.287	0.055	0.983
Gormley P, 2016 [40]	*SPINK2*/rs7684253	T vs. C	0.96 (0.94–0.97)	2.5E-09	Overall (Caucasian 22)	59,674/316078	1.153E-11	1.154E-08	1.153E-11	1.154E-08	4.525E-09	4.530E-06
Gormley P, 2016 [40]	*ZCCHC14*/rs4081947	G vs. A	1.03 (1.00–1.06)	2.5E-09	Overall (Caucasian 22)	59,674/316078	0.978	1.000	0.978	1.000	1.000	1.000
Gormley P, 2016 [40]	*HEY2*/rs1268083	C vs. T	0.96 (0.95–0.97)	5.3E-09	Overall (Caucasian 22)	59,674/316078	1.153E-11	1.154E-08	1.153E-11	1.154E-08	4.525E-09	4.530E-06
Gormley P, 2016 [40]	*WSCD1*/rs75213074	T vs. C	0.89 (0.86–0.93)	7.1E-09	Overall (Caucasian 22)	59,674/316078	2.044E-04	0.170	2.040E-04	0.170	0.015	0.937
Gormley P, 2016 [40]	*GJA1*/rs28455731	T vs. G	1.06 (1.04–1.08)	7.3E-09	Overall (Caucasian 22)	59,674/316078	9.959E-07	9.959E-04	9.959E-07	9.959E-04	1.767E-04	0.150
Gormley P, 2016 [40]	*TGFBR2*/rs6791480	T vs. C	1.04 (1.03–1.06)	7.8E-09	Overall (Caucasian 22)	59,674/316078	0.052	0.982	0.052	0.982	0.863	1.000
Gormley P, 2016 [40]	*ITPK1*/rs11624776	C vs. A	0.96 (0.94–0.97)	7.9E-09	Overall (Caucasian 22)	59,674/316078	1.153E-11	1.154E-08	1.153E-11	1.154E-08	4.525E-09	4.530E-06
Gormley P, 2016 [40]	*ADAMTSL4*/rs6693567	T vs. C	1.05 (1.03–1.06)	1.2E-08	Overall (Caucasian 22)	59,674/316078	–	–	–	–	3.476E-18	3.479E-15
Gormley P, 2016 [40]	*CCM2L*/rs144017103	T vs. C	0.85 (0.76–0.96)	1.2E-08	Overall (Caucasian 22)	59,674/316078	0.934	1.000	0.899	1.000	0.993	1.000
Gormley P, 2016 [40]	*YAP1*/rs10895275	A vs. T	1.04 (1.03–1.06)	1.6E-08	Overall (Caucasian 22)	59,674/316078	0.052	0.982	0.052	0.982	0.863	1.000
Gormley P, 2016 [40]	*MED14*/rs12845494	G vs. C	0.96 (0.95–0.97)	1.7E-08	Overall (Caucasian 22)	59,674/316078	1.153E-11	1.154E-08	1.153E-11	1.154E-08	4.525E-09	4.530E-06
Gormley P, 2016 [40]	*DOCK4*/rs10155855	T vs. A	1.08 (1.05–1.12)	2.1E-08	Overall (Caucasian 22)	59,674/316078	0.032	0.971	0.032	0.971	0.688	1.000
Gormley P, 2016 [40]	*LRRIQ3*/rs1572668	G vs. A	1.04 (1.02–1.05)	2.1E-08	Overall (Caucasian 22)	59,674/316078	8.873E-13	8.882E-10	8.873E-13	8.882E-10	4.186E-10	4.190E-07
Gormley P, 2016 [40]	*CARF*/rs138556413	G vs. A	0.88 (0.84–0.92)	2.3E-08	Overall (Caucasian 22)	59,674/316078	1.748E-05	0.017	1.733E-05	0.017	1.395E-03	0.583
Gormley P, 2016 [40]	*ARMS2*/rs2223089	C vs. G	0.93 (0.91–0.95)	3.0E-08	Overall (Caucasian 22)	59,674/316078	2.308E-08	2.310E-05	2.308E-08	2.310E-05	4.007E-06	3.995E-03
Gormley P, 2016 [40]	*IGSF9B*/rs561561	T vs. A	0.94 (0.92–0.96)	3.4E-08	Overall (Caucasian 22)	59,674/316078	8.384E-06	0.008	8.384E-06	0.008	1.254E-03	0.557
Gormley P, 2016 [40]	*MPPED2*/rs11031122	C vs. T	1.04 (1.03–1.06)	3.5E-08	Overall (Caucasian 22)	59,674/316078	0.052	0.982	0.052	0.982	0.863	1.000
Gormley P, 2016 [40]	*NOTCH4*/rs140002913	A vs. G	0.91 (0.88–0.94)	3.8E-08	Overall (Caucasian 22)	59,674/316078	1.204E-05	0.012	1.204E-05	0.012	1.229E-03	0.552
Anttila V, 2013 [29]	*PRDM16*/rs2651899	C vs. T	1.09 (1.07–1.12)	3.28E-14	Overall (Caucasian 19)	23,285/95425	4.933E-07	4.935E-04	4.933E-07	4.935E-04	6.430E-05	0.0605
Anttila V, 2013 [29]	*TSPAN2*/rs12134493	A vs. C	1.14 (1.10–1.18)	6.71E-14	Overall (Caucasian 19)	23,285/95425	9.555E-11	9.565E-08	9.538E-11	9.548E-08	1.305E-08	1.307E-05
Anttila V, 2013 [29]	*MEF2D*/rs2274316	C vs. A	1.07 (1.04–1.09)	3.14E-08	Overall (Caucasian 19)	23,285/95425	8.014E-10	8.022E-07	8.014E-10	8.022E-07	1.694E-07	1.695E-04
Anttila V, 2013 [29]	*TRPM8*/rs7577262	A vs. G	0.87 (0.84–0.90)	3.27E-13	Overall (Caucasian 19)	23,285/95425	8.231E-13	8.239E-10	8.180E-13	8.188E-10	1.259E-10	1.260E-07
Anttila V, 2013 [29]	*FHL5*/rs13208321	A vs. T	1.18 (1.13–1.24)	2.15E-12	Overall (Caucasian 19)	23,285/95425	8.181E-08	8.189E-05	6.109E-08	6.115E-05	5.781E-06	5.753E-03
Anttila V, 2013 [29]	*c7orf10*/rs4379368	T vs. C	1.11 (1.08–1.15)	1.46E-09	Overall (Caucasian 19)	23,285/95425	7.560E-06	7.511E-03	7.560E-06	7.511E-03	7.339E-04	0.424
Anttila V, 2010 [16]	*MTDH*/rs1835740	C vs. T	1.18 (1.13–1.24)	1.60E-11	Overall (Caucasian 7)	5950/50809	1.109E-06	1.109E-03	8.138E-07	8.139E-04	6.811E-05	0.064

*GWAS* genome-wide association studies, *OR* odds radio, *CI* confidence interval, *FPRP* false-positive rate probability, *BFDP* Bayesian false discovery probability

As shown in Table 1, 5 genes with 8 genetic variants from observational studies were found to be significant after excluding the overlapping data. At a prior probability of 0.05, we identified the genetic variants, *MTHFR*/rs1801133, noteworthy via FPRP estimation with a statistical power to detect OR of 1.2. Likewise, 4 genetic variants including *MTHFR*/rs1801133, *BDNF*/rs6265, *ESR1*/rs1801132 and *5-HTT*/VNTR showed noteworthy FPRP values to detect OR of 1.5. As for the statistical method BFDP, the same 4 genetic variants were noteworthy. However, when it comes to the re-analysis at a prior probability of 0.001, no noteworthy relationship between genetic variants and migraine risk could be detected via FPRP and BFDP.

Moreover, we performed subgroup analysis of observational studies based on the migraine subtypes (Table 3). In the migraine with aura subgroup, only 1 (*TNF-α*/rs1800629) and 3 (*MTHFR*/rs1801133, *ESR1*/rs1801132 and *TNF-α*/rs1800629) genetic variants was noteworthy in FPRP at the prior probability of 0.05 with a statistical power to detect an OR of 1.2 and 1.5, respectively. And all the variants with noteworthy FPRP values were also noteworthy in BFDP estimation. Similarly, none was identified to be noteworthy in FPRP and BFDP when the prior probability was 0.001. In migraine without aura, we did not observe any noteworthy SNPs. Subsequent subgroup analyses based on ethnicity were performed and displayed in Table 4. Compared with 4 noteworthy SNPs identified at the prior probability of 0.05 containing *MTHFR*/rs1801133, *BDNF*/rs6265, *ESR1*/rs1801132 and *ESR1*/rs2228480 for Caucasian population, none of candidate polymorphisms was considered to be noteworthy in non-Caucasian population.

**Table 3:** Subgroup analysis of genetic variants with statistically significance (*P*-value< 0.05) from observational studies based on migraine subtype

Author, year	Gene/variant	Comparison	OR (95%CI)	*P*-value	Subtypes	No. of cases/controls	FPRP Values at Prior probability				BFDP 0.05	BFDP 0.001
							OR 1.2		OR 1.5			
							0.05	0.001	0.05	0.001		
Liu L, 2019 [48]	*MTHFR*/rs1801133	T vs. C	1.28 (1.09–1.51)	0.003	MA	4313/28092	0.226	0.939	0.063	0.778	0.560	0.985
Liu L, 2019 [48]	*MTHFR*/rs1801133	CT + TT vs. CC	1.20 (1.00–1.44)	0.049	MA	4313/28092	0.655	0.990	0.489	0.981	0.904	0.998
Liu L, 2019 [48]	*MTHFR*/rs1801133	TT vs. CT + CC	1.46 (1.10–1.95)	0.010	MA	4313/28092	0.682	0.991	0.256	0.948	0.788	0.995
Liu L, 2019 [48]	*MTHFR*/rs1801133	TT vs. CC	1.51 (1.09–2.08)	0.012	MA	4313/28092	0.735	0.993	0.314	0.960	0.812	0.996
Terrazzino S, 2017 [44]	*BDNF*/rs6265	GA + AA vs. GG	1.22 (1.00–1.47)	0.047	MA	717/1593	0.617	0.988	0.413	0.974	0.882	0.997
Li L, 2015 [37]	*ESR1*/rs1801132	GG vs. CC	1.59 (1.17–2.15)	0.003	MA	1427/1919	0.593	0.987	0.123	0.880	0.632	0.989
Li L, 2015 [37]	*ESR1*/rs1801132	GG vs. CG + CC	1.58 (1.18–2.13)	0.002	MA	1427/1919	0.590	0.987	0.122	0.880	0.633	0.989
Chen M, 2015 [35]	*TNF-α*/rs1800629	AA+GA vs. GG	1.17 (1.05–1.30)	0.004	MA	1763/21837	0.089	0.837	0.062	0.777	0.582	0.987
Chen M, 2015 [35]	*TNF-α*/rs1800629	A vs. G	1.13 (1.03–1.24)	0.010	MA	1763/21837	0.173	0.917	0.159	0.908	0.783	0.995
Chen M, 2015 [35]	*TNF-α*/rs1800629	GA vs. GG	1.17 (1.05–1.31)	0.005	MA	1763/21837	0.115	0.906	0.110	0.866	0.694	0.992
Chen M, 2015 [35]	*NOS3*/rs1799983	TT vs. GG	1.61 (1.12–2.31)	0.010	MA	440/881	0.770	0.994	0.345	0.965	0.816	0.996
Chen M, 2015 [35]	*NOS3*/rs1799983	TT vs. GT + GG	1.50 (1.08–2.09)	0.016	MA	440/881	0.771	0.994	0.387	0.971	0.843	0.996
Liu H, 2011 [23]	*5-HTT*/VNTR	12/other	1.33 (1.01–1.75)	0.042	MA	176/629	0.774	0.994	0.496	0.981	0.891	0.998
Liu H, 2011 [23]	*5-HTT*/VNTR	12/12 vs. 12/other +other	1.58 (1.07–2.33)	0.021	MA	176/629	0.829	0.996	0.501	0.981	0.873	0.997
Schurks M, 2010 [20]	*ACE*/rs1799752	II vs. ID + DD	0.71 (0.55–0.93)	0.011	MA	1761/22310	0.667	0.991	0.266	0.950	0.801	0.995
Liu L, 2019 [48]	*MTHFR*/rs1801131	C vs. A	1.43 (1.06–1.92)	0.018	MO	159/1477	0.730	0.993	0.345	0.965	0.835	0.996
Liu L, 2019 [48]	*MTHFR*/rs1801131	CC vs. AC + AA	2.74 (1.46–5.14)	0.002	MO	159/1477	0.864	0.997	0.515	0.982	0.842	0.996
Liu L, 2019 [48]	*MTHFR*/rs1801131	CC vs. AA	2.83 (1.30–6.16)	0.009	MO	159/1477	0.916	0.998	0.752	0.994	0.991	0.998
Liu H, 2011 [23]	*5-HTT*/VNTR	12/other	1.30 (1.02–1.67)	0.037	MO	319/697	0.741	0.993	0.467	0.979	0.887	0.998
Liu H, 2011 [23]	*5-HTT*/VNTR	12/12 vs. 12/other +other	1.55 (1.11–2.16)	0.010	MO	319/697	0.737	0.993	0.302	0.958	0.801	0.995
Schurks M, 2010 [20]	*ACE*/rs1799752	II vs. ID + DD	0.84 (0.70–0.99)	0.049	MO	2853/22310	0.570	0.986	0.417	0.974	0.888	0.998

*MA* migraine with aura, *MO* migraine without aura

**Table 4:** Subgroup analysis of genetic variants with statistically significance (*P*-value< 0.05) from observational studies based on ethnicity

Author, year	Gene/variant	Comparison	OR (95%CI)	*P*-Value	Ethnicity	No. of cases/controls	FPRP Values at Prior probability				BFDP 0.05	BFDP 0.001
							OR 1.2		OR 1.5			
							0.05	0.001	0.05	0.001		
Liu L, 2019 [48]	*MTHFR*/rs1801133	T vs. C	1.18 (1.04–1.34)	0.012	Caucasian 20	9635/27592	0.253	0.947	0.169	0.915	0.766	0.994
Liu L, 2019 [48]	*MTHFR*/rs1801133	TT vs. CT + CC	1.25 (1.02–1.53)	0.035	Caucasian 20	9635/27592	0.626	0.989	0.376	0.969	0.867	0.997
Liu L, 2019 [48]	*MTHFR*/rs1801133	TT vs. CC	1.28 (1.02–1.60)	0.036	Caucasian 20	9635/27592	0.667	0.991	0.384	0.968	0.886	0.997
Dong H, 2018 [46]	*eNOS*/rs2070744	CC vs. TC + TT	1.62 (1.03–2.56)	0.04	Caucasian 4	435/344	0.881	0.997	0.665	0.991	0.908	0.998
Terrazzino S, 2017 [44]	*BDNF*/rs6265	A vs. G	1.17 (1.03–1.34)	0.014	Caucasian 5	2884/3760	0.408	0.973	0.307	0.959	0.856	0.997
Terrazzino S, 2017 [44]	*BDNF*/rs6265	AA + GA vs. GG	1.22 (1.05–1.41)	0.011	Caucasian 5	2884/3760	0.247	0.945	0.119	0.877	0.694	0.992
Cai X, 2017 [42]	*BDNF*/rs2049046	A vs. T	0.88 (0.79–0.98)	0.02	Caucasian 4	1260/1380	0.311	0.960	0.275	0.952	0.855	0.997
Cai X, 2017 [42]	*BDNF*/rs2049046	AA vs. TA + TT	0.80 (0.67–0.96)	0.02	Caucasian 4	1260/1380	0.486	0.980	0.243	0.944	0.809	0.996
Cai X, 2017 [42]	*BDNF*/rs2049046	AA vs. TT	0.78 (0.62–0.97)	0.02	Caucasian 4	1260/1380	0.637	0.989	0.345	0.965	0.853	0.997
Cai X, 2017 [42]	*BDNF*/rs2049046	AA vs. TA	0.81 (0.67–0.99)	0.03	Caucasian 4	1260/1380	0.658	0.990	0.436	0.976	0.887	0.976
Li L, 2015 [37]	*ESR1*/rs1801132	GG vs. CC	1.63 (1.20–2.22)	0.002	Caucasian 4	1693/1719	0.586	0.987	0.110	0.866	0.600	0.988
Li L, 2015 [37]	*ESR1*/rs1801132	GG vs. CG + CC	1.63 (1.21–2.21)	0.001	Caucasian 4	1693/1719	0.564	0.986	0.096	0.848	0.572	0.986
Li L, 2015 [37]	*ESR1*/rs2228480	AG vs. GG	1.19 (1.04–1.35)	0.009	Caucasian 5	1959/1826	0.192	0.926	0.116	0.873	0.696	0.992
Li L, 2015 [37]	*ESR1*/rs2228480	AA + AG vs. GG	1.17 (1.04–1.33)	0.016	Caucasian 5	1959/1826	0.323	0.962	0.237	0.942	0.821	0.996
Chen M, 2015 [35]	*TNF-α*/rs1800629	A vs. G	1.74 (1.13–2.67)	0.012	Non-Caucasian 5	985/956	0.828	0.996	0.462	0.978	0.851	0.997
Chen M, 2015 [35]	*TNF-α*/rs1800629	AA+GA vs. GG	1.82 (1.15–2.87)	0.010	Non-Caucasian 5	985/956	0.838	0.996	0.483	0.980	0.854	0.997
Chen M, 2015 [35]	*TNF-α*/rs1800629	GA vs. GG	1.78 (1.17–2.72)	0.007	Non-Caucasian 5	985/956	0.810	0.996	0.405	0.973	0.828	0.996
Chen M, 2015 [35]	*NOS3*/rs1799983	TT vs. GT + GG	1.84 (1.02–3.33)	0.043	Non-Caucasian 3	504/339	0.914	0.998	0.770	0.994	0.923	0.998
Chen M, 2015 [35]	*NOS3*/rs1799983	TT vs. GG	2.10 (1.14–3.88)	0.018	Non-Caucasian 3	504/339	0.902	0.998	0.706	0.992	0.907	0.998
Liu R, 2014 [32]	*TNF-β*/rs909253	GG vs. AG + AA	1.38 (1.04–1.84)	0.027	Non-Caucasian 3	746/717	0.759	0.994	0.428	0.975	0.869	0.997

In addition to FPRP and BFDP, we also used the Venice criteria to evaluate the credibility of the meta-analyses characterized by low, intermediate and high level (Table 1). We observed a consistency between the noteworthiness measured by FPRP and BFDP and Venice criteria score for candidate SNPs, that is, most of the noteworthy SNPs observed in our re-analysis were with high or intermediate level of evidence, with the exception of *MTHFR*/rs1801133.

As shown in Table 2, within the data extracted from the meta-analyses of GWAS, 40 genes with 47 genetic variants were statistically significant (*P* < 5 × 10^− 8^). According to the results of the re-analyses, 32 and 26 SNPs were found to be noteworthy via FPRP estimation at the statistical power to detect the OR of 1.2 with the prior probability of 0.001 and 0.000001. 34 and 26 SNPs were identified as noteworthy to detect the OR of 1.5 via FPRP. As for BFDP, 40 and 35 SNPs were noteworthy under the prior probability of 0.001 and 0.000001. Almost all noteworthy variants calculated through FPRP also showed noteworthy BFDP values. Only three variants (*YAP1*/rs10895275, *TGFBR2*/rs6791480 and *MPPED2*/ rs11031122) were noteworthy in the calculation of FPRP, but not via BFDP. In addition, we detected 9 noteworthy SNPs (*LRP1*/rs11172113, *PRDM16*/rs10218452, *FHL5*/rs67338227, *TSPAN2*/rs2078371, *PLCE1*/ rs10786156, *KCNK5*/rs10456100, *CFDP1*/rs77505915, *NRP1*/rs2506142 and *ADAMTSL4*/rs6693567) in the computation of BFDP rather than FPRP, which could be explained by the fact that, in some cases, noteworthiness could not be assessed on account of a mathematical error during the calculation of the inverse of cumulative normal distribution in FPRP. Furthermore, in the subgroup analysis, we only performed the re-analysis of migraine without aura but not migraine with aura owing to the lack of raw data. We found that all ten statistically significant variants within 8 genes were noteworthy in migraine without aura subgroup (Table 5). In addition, due to the population included in the GWAS were almost Caucasians, subgroup re-analysis based on ethnicity cannot be performed.

**Table 5:** Subgroup analysis results of genetic variants with statistically significance (*P*-value< 5*10^−8^) from GWAS studies based on migraine subtype

Author, year	Gene/variant	Comparison	OR (95%CI)	*P*-value	Subtypes	No. of cases/controls	FPRP Values at Prior probability				BFDP 0.001	BFDP 0.000001
							OR 1.2		OR 1.5			
							0.001	0.000001	0.001	0.000001		
Gormley P, 2016 [40]	*LRP1*/rs11172113	C vs. T	0.85 (0.82–0.89)	4.3E-16	MO	8348/139622	5.365E-09	5.370E-06	4.295E-09	4.300E-06	4.578E-07	4.580E-04
Gormley P, 2016 [40]	*FHL5*/rs7775721	T vs. A	1.15 (1.11–1.20)	1.1E-12	MO	8348/139622	1.253E-07	1.254E-04	1.222E-07	1.223E-04	1.210E-05	0.012
Gormley P, 2016 [40]	*ASTN2*/rs6478241	G vs. A	1.14 (1.09–1.18)	1.2E-10	MO	8348/139622	9.555E-11	9.565E-08	9.538E-11	9.548E-08	1.305E-08	1.307E-05
Gormley P, 2016 [40]	*TRPM8*/rs6724624	G vs. C	0.86 (0.82–0.90)	1.1E-09	MO	8348/139622	8.654E-08	8.662E-05	7.899E-08	7.906E-05	7.681E-06	7.630E-03
Gormley P, 2016 [40]	*PHACTR1*/rs9349379	G vs. A	0.88 (0.85–0.92)	2.1E-09	MO	8348/139622	1.748E-05	0.017	1.733E-05	0.017	1.395E-03	0.583
Gormley P, 2016 [40]	*FGF6*/rs1024905	A vs. G	1.12 (1.08–1.16)	2.5E-09	MO	8348/139622	2.451E-07	2.453E-04	2.451E-07	2.453E-04	2.680E-05	0.026
Gormley P, 2016 [40]	*TSPAN2*/rs2078371	C vs. T	1.18 (1.12–1.25)	7.4E-09	MO	8348/139622	2.525E-05	0.025	1.808E-05	0.018	1.276E-03	0.561
Anttila V, 2013 [29]	*TRPM8*/rs6741751	A vs. G	0.80 (0.75–0.86)	8.64E-11	MO	7107/69427	1.094E-05	0.011	1.469E-06	1.469E-03	1.143E-04	0.103
Anttila V, 2013 [29]	*LRP1*/rs11172113	C vs. T	0.87 (0.84–0.91)	9.96E-11	MO	7107/69427	1.300E-06	1.300E-03	1.260E-06	1.260E-03	1.117E-04	0.101
Anttila V, 2013 [29]	*PHACTR1*/rs9349379	G vs. A	0.86 (0.82–0.90)	2.81E-10	MO	7107/69427	8.654E-08	8.662E-05	7.899E-08	7.906E-05	7.681E-06	7.630E-03
Anttila V, 2013 [29]	*FHL5*/rs11759769	A vs. G	1.18 (1.13–1.24)	1.58E-12	MO	7107/69427	8.181E-08	8.189E-05	6.109E-08	6.115E-05	5.781E-06	5.753E-03
Anttila V, 2013 [29]	*MMP16*/rs10504861	T vs. C	0.86 (0.81–0.90)	1.17E-08	MO	7107/69427	8.654E-08	8.662E-05	7.899E-08	7.906E-05	7.681E-06	7.630E-03

*GWAS* genome-wide association studies, *MO* migraine without aura

In order to provide readers with the predictive potential of the migraine risk, we calculated the joint PAR which was recently argued to be more credible than *P*-values or ORs by combining 36 noteworthy variants via FPRP or BFDP. And the value of joint PAR in our study was 44.2%, indicating that these involved SNPs together may attribute to an obvious increment in the risk of migraine and this method may be a useful way for screening migraine susceptibility and identify at-risk populations (Table 6).

**Table 6:** Combination of genetic polymorphisms to predict risk of migraine. Calculation of joint PAR% using the SNPs showing noteworthiness during the computing of BFDP (BFDP < 0.8) or FPRP (FPRP< 0.2) at a statistical power to detect the OR of 1.5

Author, year	Gene/variant	Comparison	MAF	OR (95%CI)	*P*-value	Ethnicity	No. of cases/controls	PAR (%)	Joint PAR (%)
Chang X, 2018 [45]	*NMUR2*/rs72793414	A vs. G	0.1082	2.44 (1.85–3.23)	3.81E-10	Overall 2	1212/13494	13.4804396578	44.2094776354
Gormley P, 2016 [40]	*LRP1*/rs11172113	C vs. T	0.3894	0.90 (0.89–0.91)	5.6E-49	Caucasian 22	59,674/316078	4.0517761638	
Gormley P, 2016 [40]	*PRDM 16*/rs10218452	G vs. A	0.2264	1.11 (1.10–1.13)	5.3E-38	Caucasian 22	59,674/316078	2.4298861162	
Gormley P, 2016 [40]	*FHL5*/rs67338227	T vs. A	0.0220	1.09 (1.08–1.11)	2.0E-27	Caucasian 22	59,674/316078	0.1976087347	
Gormley P, 2016 [40]	*TSPAN2*/rs2078371	C vs. T	0.1252	1.11 (1.09–1.13)	4.1E-24	Caucasian 22	59,674/316078	1.3584908638	
Gormley P, 2016 [40]	*PHACTR1*/rs9349379	G vs. A	0.3774	0.93 (0.92–0.95)	5.8E-22	Caucasian 22	59,674/316078	2.7134848426	
Gormley P, 2016 [40]	*MEF2D*/rs1925950	A vs. G	0.4277	1.07 (1.06–1.09)	9.1E-22	Caucasian 22	59,674/316078	2.9068711836	
Gormley P, 2016 [40]	*SLC24A3*/rs4814864	C vs. G	0.3021	1.07 (1.06–1.09)	2.2E-19	Caucasian 22	59,674/316078	2.0709065394	
Gormley P, 2016 [40]	*FGF6*/rs1024905	A vs. G	0.3165	1.06 (1.04–1.08)	2.1E-17	Caucasian 22	59,674/316078	1.8636100452	
Gormley P, 2016 [40]	*C7orf10*/rs186166891	T vs. A	0.1631	1.09 (1.07–1.12)	9.7E-16	Caucasian 22	59,674/316078	1.4466644131	
Gormley P, 2016 [40]	*PLCE1*/rs10786156	G vs. C	0.4852	0.95 (0.94–0.96)	2.0E-14	Caucasian 22	59,674/316078	2.4863180765	
Gormley P, 2016 [40]	*KCNK5*/rs10456100	T vs. C	0.1813	1.06 (1.04–1.07)	6.9E-13	Caucasian 22	59,674/316078	1.0760942468	
Gormley P, 2016 [40]	*HPSE2*/rs12260159	A vs. G	0.1673	0.92 (0.89–0.94)	3.2E-10	Caucasian 22	59,674/316078	1.3565561475	
Gormley P, 2016 [40]	*CFDP1*/rs77505915	A vs. T	0.4898	1.05 (1.03–1.06)	3.3E-10	Caucasian 22	59,674/316078	2.3904576911	
Gormley P, 2016 [40]	*RNF213*/rs17857135	C vs. T	0.2115	1.06 (1.04–1.08)	5.2E-10	Caucasian 22	59,674/316078	1.2530981840	
Gormley P, 2016 [40]	*NRP1*/rs2506142	G vs. A	0.1865	1.06 (1.04–1.07)	1.5E-09	Caucasian 22	59,674/316078	1.1066169563	
Gormley P, 2016 [40]	*GPR149*/rs13078967	C vs. A	0.0110	0.87 (0.83–0.91)	1.8E-09	Caucasian 22	59,674/316078	0.1432047828	
Gormley P, 2016 [40]	*SPINK2*/rs7684253	T vs. C	0.4499	0.96 (0.94–0.97)	2.5E-09	Caucasian 22	59,674/316078	1.8325790934	
Gormley P, 2016 [40]	*HEY2*/rs1268083	C vs. T	0.4419	0.96 (0.95–0.97)	5.3E-09	Caucasian 22	59,674/316078	1.7994063059	
Gormley P, 2016 [40]	*WSCD1*/rs75213074	T vs. C	0.0094	0.89 (0.86–0.93)	7.1E-09	Caucasian 22	59,674/316078	0.1035070263	
Gormley P, 2016 [40]	*GJA1*/rs28455731	T vs. G	0.1302	1.06 (1.04–1.08)	7.3E-09	Caucasian 22	59,674/316078	0.7751445706	
Gormley P, 2016 [40]	*ITPK1*/rs11624776	C vs. A	0.2278	0.96 (0.94–0.97)	7.9E-09	Caucasian 22	59,674/316078	0.9195792057	
Gormley P, 2016 [40]	*ADAMTSL4*/rs6693567	T vs. C	0.3037	1.05 (1.03–1.06)	1.2E-08	Caucasian 22	59,674/316078	1.4957864823	
Gormley P, 2016 [40]	*MED14*/rs12845494	G vs. C	0.4114	0.96 (0.95–0.97)	1.7E-08	Caucasian 22	59,674/316078	1.6731330779	
Gormley P, 2016 [40]	*LRRIQ3*/rs1572668	G vs. A	0.4930	1.04 (1.02–1.05)	2.1E-08	Caucasian 22	59,674/316078	1.9338641980	
Gormley P, 2016 [40]	*CARF*/rs138556413	G vs. A	0.0104	0.88 (0.84–0.92)	2.3E-08	Caucasian 22	59,674/316078	0.1249559450	
Gormley P, 2016 [40]	*ARMS2*/rs2223089	C vs. G	0.1288	0.93 (0.91–0.95)	3.0E-08	Caucasian 22	59,674/316078	0.9098027819	
Gormley P, 2016 [40]	*IGSF9B*/rs561561	T vs. A	0.0827	0.94 (0.92–0.96)	3.4E-08	Caucasian 22	59,674/316078	0.4986744225	
Gormley P, 2016 [40]	*NOTCH4*/rs140002913	A vs. G	0.1150	0.91 (0.88–0.94)	3.8E-08	Caucasian 22	59,674/316078	1.0458242813	
Anttila V. 2013 [29]	*PRDM16*/rs2651899	C vs. T	0.4708	1.09 (1.07–1.12)	3.28E-14	Caucasian 19	23,285/95425	4.0649595346	
Anttila V. 2013 [29]	*TSPAN2*/rs12134493	A vs. C	0.0709	1.14 (1.10–1.18)	6.71E-14	Caucasian 19	23,285/95425	0.9828442876	
Anttila V. 2013 [29]	*MEF2D*/rs2274316	C vs. A	0.4289	1.07 (1.04–1.09)	3.14E-08	Caucasian 19	23,285/95425	2.9147892814	
Anttila V. 2013 [29]	*TRPM8*/rs7577262	A vs. G	0.2356	0.87 (0.84–0.90)	3.27E-13	Caucasian 19	23,285/95425	3.1595713513	
Anttila V. 2013 [29]	*FHL5*/rs13208321	A vs. T	0.2949	1.18 (1.13–1.24)	2.15E-12	Caucasian 19	23,285/95425	5.0406331131	
Anttila V. 2013 [29]	*c7orf10*/rs4379368	T vs. C	0.1961	1.11 (1.08–1.15)	1.46E-09	Caucasian 19	23,285/95425	2.1115517179	
Anttila V. 2013 [29]	*MTDH*/rs1835740	C vs. T	0.3510	1.18 (1.13–1.24)	1.60E-11	Caucasian 7	5950/50809	5.9425497094	

*MAF* minor allelic frequency, *PAR* population attributable risk

**Fig. 2:**
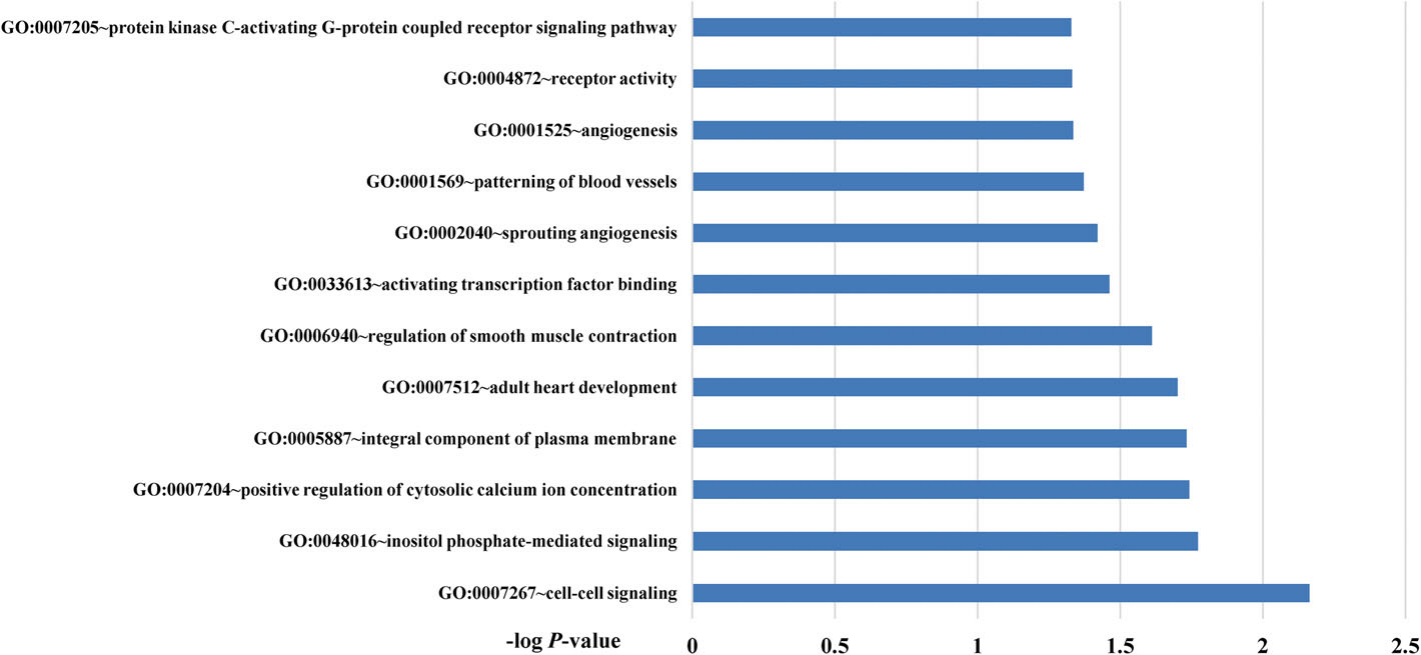
Gene ontology enrichment analysis of migraine. The cut-off *P*-value for this analysis was 0.05 (−log *P*-value = 1.3)

To explore the potential biology mechanism for migraine, GO enrichment analysis was performed. As shown in Fig. 2, twelve GO terms were identified including “cell-cell signaling” (GO: 0007267), “inositol phosphate-mediated signaling” (GO:0048016), “positive regulation of cytosolic calcium ion concentration” (GO:0007204), “integral component of plasma membrane” (GO:0005887), “adult heart development” (GO:0007512), “regulation of smooth muscle contraction” (GO:0006940), “activating transcription factor binding” (GO:0033613), “sprouting angiogenesis” (GO:0002040), “patterning of blood vessels” (GO:000156), “angiogenesis” (GO:0001525), “receptor activity” (GO:0004872) and “protein kinase C-activating G-protein coupled receptor signaling pathway” (GO:0007205). To detect the interactive relationships and potential hub nodes, PPI network of all genes with noteworthy SNPs were constructed and displayed in Additional file 3: Figure S1. Moreover, when degree ≥7 as the cut-off criterion, 5 genes (*MEF2D, TSPAN2, PHACTR1, TRPM8* and *PRDM16*) were selected as hub genes by using CytoHubba software.

## Discussion

Despite the serious impact of migraine on human health and its burden on public, understanding of the pathogenesis, valid prevention and treatment remains limited [49, 50]. Recent years, the genetic component of migraine has received widespread attention. Moreover, a growing number of meta-analyses from observational studies and GWAS were performed and further identified numerous significant genetic variants, which displayed important insights into the mechanisms underlying migraine development. However, due to false-positive possibility in meta-analyses, we performed the first re-analysis of meta-analyses of genetic association studies in migraine in hope of finding noteworthy associations. We used Bayesian methods and Venice criteria to evaluate the credibility of genetic associations. In this work, we synthesized all relevant data from the meta-analyses which detected the association between genetic polymorphisms and migraine risk before 31 July 2019.

As for the candidate genes, we found 4 genetic variants noteworthy at prior probability of 0.05 which were consistent with the results of Venice criteria. However, when we raised the prior probability standard from 0.05 to 0.001, we did not find any gene noteworthy under FPRP or BFDP, which indicated that the results of observational studies should be interpreted with caution. In comparison to the observational studies, according to GWAS, out of the 47 significant genetic variants, 36 were considered to be noteworthy at prior probability of 0.000001 via FPRP or BFDP. Most significant variants identified in GWAS remained noteworthy at the prior probability of 0.000001 demonstrated that the results from GWAS were more reliable.

We also detected the pathways involved in migraine by conducting functional enrichment analysis and further explore the possible molecular mechanisms. We found several significant pathways (cell-cell signaling, inositol phosphate-mediated signaling, positive regulation of cytosolic calcium ion concentration, integral component of plasma membrane and adult heart development, etc.) and 5 hub genes (*MEF2D*, *TSPAN2*, *PHACTR1*, *TRPM8* and *PRDM16*) which were considered to play vital roles in migraine occurrence.

To date, the pathophysiology of migraine is partially understood that the headache of migraine is associated with activation and sensitization of trigeminovascular system [51, 52]. This hypothesis is based on a fact that migraine attack was originated from the activation of nociceptors that are initialed from trigeminal ganglion and innervate arachnoid, pial, blood vessels of dural, large cerebral arteries and sinuses [53]. These activated nociceptors released some inflammatory mediators and vasoactive neuropeptides such as calcitonin gene-related peptide (CGRP), neurokinin A and substance P causing vasodilation of dural and pial vessels and neurogenic inflammation which finally results in central sensitization causing headache of migraine to some extent [53–55].

*PLCE1* and *NMUR2*, whose variants showed noteworthy association with migraine susceptibility, were found to be involved in two pathways called “positive regulation of cytosolic calcium ion concentration” and “inositol phosphate-mediated signaling” detected in our study. *PLCE1* is a gene encodes an enzyme called phospholipase C that promotes the generation of inositol triphosphate (IP3) and further direct the calcium mobilization by initiate the release of calcium ion stored in the endoplasmic reticulum through IP3 receptor leading to the increment of cytosolic calcium concentration in neurons [56, 57]. As for *NMUR2*, an impaired pain response was observed in *NMUR2*-deficient mice, supporting the hypothesis that nociceptive effects may be partially mediated through NMUR2 [58]. Similar with PLCE1, NMUR2 is abundantly expressed in the central nervous system and considered as a regulator for intracellular calcium mobilization via IP3 as well [59]. Furthermore, the increasing of cytosolic calcium may cause the release of CGRP that finally results in migraine development [60, 61].

Within the hub genes detected in our study, *TRPM8* gene was found to play an important role in the pathophysiology of migraine. As a cation channel, TRPM8 is characterized as a cold temperature detector firstly [62, 63]. However, recently, it is also discovered as a parameter for ongoing persistent pain with several evidence. *TRPM8* null mice showed a significant decrease in the injury-induced response [64]. On the opposite, the activation of TRPM8 can lead to the depolarization for nerve endings and afferent impulse into central nervous system. Moreover, these activated neurons by noxious cold temperature have properties similar with nociceptors [65]. Mechanistically, a functional linkage was observed between TRPM8 and CGRP, that is, CGRP release was deficient in neurons without TRPM8 triggering and the release of CGRP was closely related to neurogenic inflammation and future migraine occurrence [66].

As for *PHACTR1*, it might be involved in migraine attack by regulating vasomotor tone. Concretely, PHACTR1 could bind with protein phosphatase 1 (PP1) and its gene silencing has been demonstrated to decrease the activity of PP1 [67]. In arteries, PP1 may play an important role in vasomotor tone by mediating calcium cycling and contractility in smooth muscle cells [68]. Also, PHACTR1 could regulate dendritic morphology and synaptic activity by interacting with PP1 in nervous system, and was further thought to be implicated with the pathophysiology of migraine [69, 70].

Different with the hub genes mentioned above, PRDM16, a zinc-finger nuclear protein, works as an important activator of brown adipogenesis, and depletion of *PRDM16* may lead to a significant loss in brown adipocyte identity [71]. Recently, brown adipose tissue has been in the focus of metabolism research, which can dissipate energy by the regulation of uncoupling protein-1, increase fatty acid oxidation and heat production and counteract obesity [72, 73]. Moreover, *PRDM16* rs2651899 is an intron variant that may affect the splicing of *PRDM16* and its downstream regulatory elements, reduce PRDM16 expression and thus increase body mass index [74]. Large cohort studies suggest that obesity is a risk factor for migraine even after adjusting for comorbidities [75]. In obese individuals, the expression of many inflammatory mediators was increased including CGRP and interleukins, which in turn could cause central sensitization in migraine pathophysiology [76, 77]. In addition, Obesity is a state of sympathetic activation, that may contribute to increase in migraine attack [78].

For other hub genes for migraine, TSPAN2 is highly expressed in oligodendrocyte cell lines and may regulate the differentiation process of oligodendrocytes to myelin-forming glia [79]. As a transcription factor in neurons, MEF2D is concerned to be involved in neurogenesis, neuronal survival and differentiation by controlling MEF2D-dependent gene transcription [80]. However, up till now, the potential role of TSPAN2 and MEF2D in migraine development still remains unknown. Further functional studies are required to explore the underlying biological mechanisms.

Although our study included a large number of relevant articles on migraine, some limitations should be acknowledged in our re-analysis. First, most of original studies did not consider the potential confounders such as gene-gene and gene-environment interactions. Second, there exist some inherent methodological flaws in observational studies, such as selection bias, publication bias and small sample size. Although Venice criteria were applied, these potential biases were difficult to measure. Additionally, owing to the inadequate raw data, Venice Score cannot be applied for GWAS. Third, the GWAS involved in our report were mostly performed in Caucasian population, thus, the results of which was restricted to be applied in diverse populations. Also, GWAS included in our study were lack of subgroup analysis on migraine subtype. Thus, we could only study the noteworthiness of GWAS SNPs in migraine without aura subgroup. Lastly, we constructed a PPI network to explore the underlying biological mechanism for migraine. However, the criteria for PPI construction was relatively subjective, so the result of PPI might need to be interpreted with caution.

## Conclusion

The current findings identify several noteworthy variants for migraine susceptibility. We hope this field synopsis and systematic re-analysis would help identify novel genetic biomarkers and potential therapeutic target for migraine.

## Supplementary information

**Additional file 1: Table S1.** Overall summary of results from meta-analyses of observation studies on risk of migraine (including duplicates, statistically significant and non-significant results).

**Additional file 2: Table S2.** Overall summary of results from meta-analyses of GWAS on risk of migraine (including duplicates, statistically significant and non-significant results).

**Additional file 3: Figure S1.** Protein-protein interaction network of noteworthy genes related with migraine. The active interaction sources included text mining, experiments, databases as well as co-expression. MCL clustering method was used with the inflation parameter setting at 3.

## Data Availability

The datasets supporting the conclusions of this article are included within the article.
