# Supplementary material for: Genetic variants in migraine: a field synopsis and systematic re-analysis of meta-analyses
Source: J Headache Pain. 2020 Feb 11;21(1):13. doi: 10.1186/s10194-020-01087-5 (PMC7011260; doi:10.1186/s10194-020-01087-5)
Supplement: Supplementary file 1 — Additional file 1: Table S1. Overall summary of results from meta-analyses of observation studies on risk of migraine (including duplicates, statistically significant and non-significant results). [file 10194_2020_1087_MOESM1_ESM.doc]

**Table S1. Overall summary of results from meta-analyses of observation studies on risk of migraine (including duplicates, statistically significant and non-significant results).**

| Author, Year | Gene, Variant | Comparisons | OR (95%CI) | *P*-value | Ethnicity | Subgroup analysis on migraine subtypes | No. of case/control | *I*^2^ (%) | *I*^2^ (*P*) | Egger (*P*-value) | Reference |
| --- | --- | --- | --- | --- | --- | --- | --- | --- | --- | --- | --- |
| Liu L, 2019 | *MTHFR*/rs180113 | T vs. C | 1.19 (1.06-1.33) | 0.004 | Overall (Caucasian 20, Asian 6) | Yes | 10228/28608 | 81.3 | 0.000 | 0.006 | [48] |
| Liu L, 2019 | *MTHFR*/rs180113 | TT vs. CT + CC | 1.29 (1.06-1.56) | 0.010 | Overall (Caucasian 20, Asian 6) | Yes | 10228/28608 | 62.5 | — | 0.001 | [48] |
| Liu L, 2019 | *MTHFR*/rs180113 | TT + CT vs. CC | 1.17 (1.02-1.35) | 0.027 | Overall (Caucasian 20, Asian 6) | Yes | 10228/28608 | 77.3 | — | 0.045 | [48] |
| Liu L, 2019 | *MTHFR*/rs180113 | TT vs. CC | 1.32 (1.07-1.64) | 0.011 | Overall (Caucasian 20, Asian 6) | Yes | 10228/28608 | 67.9 | — | 0.002 | [48] |
| Liu L, 2019 | *MTHFR*/rs180113 | CT vs. CC | 1.09 (0.96-1.24) | 0.163 | Overall (Caucasian 20, Asian 6) | Yes | 10228/28608 | 68.1 | — | 0.192 | [48] |
| Liu L, 2019 | *MTHFR*/A1298C | C vs. A | 1.17 (0.98-1.40) | 0.088 | Overall (Caucasian 4, Asian 1) | Yes | 1368/1477 | 41.6 | — | — | [48] |
| Liu L, 2019 | *MTHFR*/A1298C | CC vs. AC + AA | 1.82 (1.09-3.04) | 0.022 | Overall (Caucasian 4, Asian 1) | Yes | 1368/1477 | 63.9 | — | — | [48] |
| Liu L, 2019 | *MTHFR*/A1298C | CC + AC vs. AA | 1.02 (0.88-1.18) | 0.839 | Overall (Caucasian 4, Asian 1) | Yes | 1368/1477 | 0 | — | — | [48] |
| Liu L, 2019 | *MTHFR*/A1298C | CC vs. AA | 1.78 (1.03-3.07) | 0.038 | Overall (Caucasian 4, Asian 1) | Yes | 1368/1477 | 62.4 | — | — | [48] |
| Liu L, 2019 | *MTHFR*/A1298C | AC vs. AA | 0.94 (0.81-1.11) | 0.474 | Overall (Caucasian 4, Asian 1) | Yes | 1368/1477 | 0 | — | — | [48] |
| Gao X, 2018 | *GRIA1*/rs548294 | A vs. G | 1.10 (0.84-1.43) | 0.49 | Overall (Caucasian 3, Asian 1) | No | 2236/2548 | 79 | 0.003 | — | [47] |
| Gao X, 2018 | *GRIA1*/rs548294 | AA vs. AG + GG | 1.15 (0.79-1.66) | 0.46 | Overall (Caucasian 3, Asian 1) | No | 1118/1274 | 56 | 0.08 | — | [47] |
| Gao X, 2018 | *GRIA1*/rs548294 | AA + AG vs. GG | 1.11 (0.81-1.54) | 0.52 | Overall (Caucasian 3, Asian 1) | No | 1118/1274 | 74 | 0.009 | — | [47] |
| Gao X, 2018 | *GRIA1*/rs548294 | AA vs. GG | 1.19 (0.72-1.97) | 0.49 | Overall (Caucasian 3, Asian 1) | No | 621/713 | 73 | 0.01 | — | [47] |
| Gao X, 2018 | *GRIA1*/rs548294 | AG vs. GG | 1.08 (0.82-1.42) | 0.58 | Overall (Caucasian 3, Asian 1) | No | 960/1117 | 59 | 0.06 | — | [47] |
| Gao X, 2018 | *GRIA1*/rs2195450 | T vs. C | 1.27 (0.91-1.77) | 0.16 | Overall (Caucasian 3, Asian 1) | No | 2174/2556 | 83 | 0.001 | — | [47] |
| Gao X, 2018 | *GRIA1*/rs2195450 | TT vs. CT + CC | 1.24 (0.62-2.46) | 0.54 | Overall (Caucasian 3, Asian 1) | No | 1087/1278 | 78 | 0.003 | — | [47] |
| Gao X, 2018 | *GRIA1*/rs2195450 | TT + CT vs. CC | 1.29 (0.96-1.74) | 0.09 | Overall (Caucasian 3, Asian 1) | No | 1087/1278 | 66 | 0.03 | — | [47] |
| Gao X, 2018 | *GRIA1*/rs2195450 | TT vs. CC | 1.30 (0.64-2.62) | 0.46 | Overall (Caucasian 3, Asian 1) | No | 744/900 | 77 | 0.004 | — | [47] |
| Gao X, 2018 | *GRIA1*/rs2195450 | CT vs. CC | 1.23 (1.02-1.48) | 0.03 | Overall (Caucasian 3, Asian 1) | No | 963/1167 | 46 | 0.14 | — | [47] |
| Dong H, 2018 | *eNOS*/rs2070744 | C vs. T | 1.30 (0.91-1.85) | 0.15 | Overall (Caucasian 4, Non-Caucasian 2) | Yes | 763/560 | 76 | — | 0.452 | [46] |
| Dong H, 2018 | *eNOS*/rs2070744 | CC vs. TC + TT | 1.27 (0.90-1.80) | 0.17 | Overall (Caucasian 4, Non-Caucasian 2) | Yes | 763/560 | 18 | 0.29 | — | [46] |
| Dong H, 2018 | *eNOS*/rs2070744 | CC + CT vs. TT | 1.66 (0.88-3.13) | 0.12 | Overall (Caucasian 4, Non-Caucasian 2) | Yes | 763/560 | 86 | — | — | [46] |
| Terrazzino, S, 2017 | *BDNF*/rs6265 | A vs. G | 1.17 (1.03-1.34) | 0.014 | Caucasian 5 | Yes | 2884/3760 | 0 | 0.60 | 0.75 | [44] |
| Terrazzino, S, 2017 | *BDNF*/rs6265 | AA vs. GA + GG | 1.18 (0.79-1.76) | 0.42 | Caucasian 5 | Yes | 1442/1880 | 0 | 0.95 | 0.90 | [44] |
| Terrazzino, S, 2017 | *BDNF*/rs6265 | AA + GA vs. GG | 1.22 (1.05-1.41) | 0.011 | Caucasian 5 | Yes | 1442/1880 | 0 | 0.63 | 0.72 | [44] |
| Liao Y, 2017 | *COMT*/rs4680 | Met vs. Val | 0.97 (0.78-1.21) | 0.805 | Overall (Caucasian 2, Asian 3) | No | 979/1870 | 54.9 | 0.065 | 0.868 | [43] |
| Liao Y, 2017 | *COMT*/rs4680 | Met/Met + Met/Val vs. Val/Val | 1.05 (0.75-1.48) | 0.773 | Overall (Caucasian 2, Asian 3) | No | 979/1870 | 58.5 | 0.047 | — | [43] |
| Liao Y, 2017 | *COMT*/rs4680 | Met/Met vs. Met/Val + Val/Val | 0.88 (0.71-1.09) | 0.246 | Overall (Caucasian 2, Asian 3) | No | 979/1870 | 29.7 | 0.224 | — | [43] |
| Liao Y, 2017 | *COMT*/rs4680 | Met/Met vs. Met/Val vs. Val/Val | 0.97 (0.77-1.23) | 0.817 | Overall (Caucasian 2, Asian 3) | No | 979/1870 | 58.2 | 0.048 | — | [43] |
| Cai X, 2017 | *BDNF*/rs6265 | G vs. A | 0.86 (0.76-0.99) | 0.03 | Caucasian 5 | No | 1353/1520 | 0 | 0.72 | — | [42] |
| Cai X, 2017 | *BDNF*/rs6265 | GG vs. GA + AA | 0.84 (0.72-0.98) | 0.03 | Caucasian 5 | No | 1353/1520 | 0 | 0.78 | — | [42] |
| Cai X, 2017 | *BDNF*/rs6265 | GG + GA vs. AA | 0.85 (0.56-1.28) | 0.42 | Caucasian 5 | No | 1353/1520 | 0 | 0.94 | — | [42] |
| Cai X, 2017 | *BDNF*/rs6265 | GG vs. AA | 0.81 (0.53-1.22) | 0.31 | Caucasian 5 | No | 1353/1520 | 0 | 0.92 | — | [42] |
| Cai X, 2017 | *BDNF*/rs6265 | GA vs. AA | 0.85 (0.72-1.00) | 0.04 | Caucasian 5 | No | 1353/1520 | 0 | 0.84 | — | [42] |
| Cai X, 2017 | *BDNF*/rs2049046 | A vs. T | 0.88 (0.79-0.98) | 0.02 | Caucasian 4 | No | 1260/1380 | 0 | 0.41 | — | [42] |
| Cai X, 2017 | *BDNF*/rs2049046 | AA vs. TA + TT | 0.80 (0.67-0.96) | 0.02 | Caucasian 4 | No | 1260/1380 | 0 | 0.72 | — | [42] |
| Cai X, 2017 | *BDNF*/rs2049046 | AA + TA vs. TT | 0.89 (0.75-1.06) | 0.18 | Caucasian 4 | No | 1260/1380 | 29 | 0.24 | — | [42] |
| Cai X, 2017 | *BDNF*/rs2049046 | AA vs. TT | 0.78 (0.62-0.97) | 0.02 | Caucasian 4 | No | 1260/1380 | 0 | 0.40 | — | [42] |
| Cai X, 2017 | *BDNF*/rs2049046 | AA vs. TA | 0.81 (0.67-0.99) | 0.03 | Caucasian 4 | No | 1260/1380 | 0 | 0.76 | — | [42] |
| Wan D, 2016 | *ACE*/rs1799752 | I vs. D | 0.95 (0.88-1.03) | — | Overall (Caucasian 4, Asian 4,Turky 4) | Yes | 6979/22767 | 30.7 | 0.146 | — | [41] |
| Wan D, 2016 | *ACE*/rs1799752 | II vs. DI+DD | 0.85 (0.70-1.03) | — | Overall (Caucasian 4, Asian 4,Turky 4) | Yes | 6979/22767 | 65.9 | 0.001 | — | [41] |
| Wan D, 2016 | *ACE*/rs1799752 | II+DI vs. DD | 1.00 (0.94-1.07) | — | Overall (Caucasian 4, Asian 4,Turky 4) | Yes | 6979/22767 | 0 | 0.780 | — | [41] |
| Wan D, 2016 | *ACE*/rs1799752 | II vs. DD | 0.85 (0.68-1.05) | — | Overall (Caucasian 4, Asian 4,Turky 4) | Yes | 6979/22767 | 55.2 | 0.011 | — | [41] |
| Wan D, 2016 | *ACE*/rs1799752 | DI vs. DD | 1.03 (0.96-1.10) | — | Overall (Caucasian 4, Asian 4,Turky 4) | Yes | 6979/22767 | 0 | 0.653 | — | [41] |
| Wan D, 2016 | *ACE*/rs1799752 | II vs. DI | 0.83 (0.67-1.03) | — | Overall (Caucasian 4, Asian 4,Turky 4) | Yes | 6979/22767 | 66.9 | 0.00 | — | [41] |
| Li L, 2015 | *ESR1*/rs1801132 | GG vs. CC | 1.51 (1.15-1.99) | — | Overall (Caucasian 4, Asian 1) | Yes | 2027/1919 | 15.9 | 0.313 | — | [37] |
| Li L, 2015 | *ESR1*/rs1801132 | CG vs. CC | 1.02 (0.90-1.16) | — | Overall (Caucasian 4, Asian 1) | Yes | 2027/1919 | 0 | 0.763 | 0.686 | [37] |
| Li L, 2015 | *ESR1*/rs1801132 | GG + CG vs. CC | 1.06 (0.94-1.19) | — | Overall (Caucasian 4, Asian 1) | Yes | 2027/1919 | 0 | 0.873 | — | [37] |
| Li L, 2015 | *ESR1*/rs1801132 | GG vs. CG + CC | 1.52 (1.16-1.98) | — | Overall (Caucasian 4, Asian 1) | Yes | 2027/1919 | 31.5 | 0.212 | — | [37] |
| Li L, 2015 | *ESR1*/rs2228480 | AA vs. GG | 1.25 (0.80-1.95) | — | Overall (Caucasian 5, Asian 1) | Yes | 2293/2026 | 54.2 | 0.053 | — | [37] |
| Li L, 2015 | *ESR1*/rs2228480 | AG vs. GG | 1.14 (1.01-1.28) | — | Overall (Caucasian 5, Asian 1) | Yes | 2293/2026 | 18.9 | 0.290 | 0.130 | [37] |
| Li L, 2015 | *ESR1*/rs2228480 | AA + AG vs. GG | 1.13 (1.00-1.26) | — | Overall (Caucasian 5, Asian 1) | Yes | 2293/2026 | 29.2 | 0.216 | — | [37] |
| Li L, 2015 | *ESR1*/rs2228480 | AA vs. AG + GG | 1.19 (0.90-1.57) | — | Overall (Caucasian 5, Asian 1) | Yes | 2293/2026 | 35.6 | 0.170 | — | [37] |
| Li L, 2015 | *ESR1*/rs2234693 | TT vs. CC | 1.15 (0.82-1.61) | — | Overall (Caucasian 1, Asian 2) | No | 606/446 | 38.7 | 0.196 | — | [37] |
| Li L, 2015 | *ESR1*/rs2234693 | CT vs. CC | 1.07 (0.86-1.34) | — | Overall (Caucasian 1, Asian 2) | No | 606/446 | 0 | 0.721 | — | [37] |
| Li L, 2015 | *ESR1*/rs2234693 | TT + CT vs. CC | 1.06 (0.88-1.29) | — | Overall (Caucasian 1, Asian 2) | No | 606/446 | 0 | 0.646 | — | [37] |
| Li L, 2015 | *ESR1*/rs2234693 | TT vs. CT + CC | 1.06 (0.77-1.44) | — | Overall (Caucasian 1, Asian 2) | No | 606/446 | 49.0 | 0.141 | — | [37] |
| Chen M, 2015 | *TNF-α*/rs1800629 | A vs. G | 1.11 (0.83-1.47) | — | Overall (Caucasian 6, Non-Caucasian 5) | Yes | 6682/22591 | 84.8 | 0.000 | 0.699 | [35] |
| Chen M, 2015 | *TNF-α*/rs1800629 | AA vs. GA+GG | 0.92 (0.77-1.11) | — | Overall (Caucasian 6, Non-Caucasian 5) | Yes | 6682/22591 | 33.1 | 0.143 | — | [35] |
| Chen M, 2015 | *TNF-α*/rs1800629 | AA+GA vs. GG | 1.14 (0.84-1.55) | — | Overall (Caucasian 6, Non-Caucasian 5) | Yes | 6682/22591 | 83.2 | 0.000 | — | [35] |
| Chen M, 2015 | *TNF-α*/rs1800629 | AA vs. GG | 0.84 (0.45-1.56) | — | Overall (Caucasian 6, Non-Caucasian 5) | Yes | 6682/22591 | 41.7 | 0.080 | — | [35] |
| Chen M, 2015 | *TNF-α*/rs1800629 | GA vs. GG | 1.15 (0.86-1.55) | — | Overall (Caucasian 6, Non-Caucasian 5) | Yes | 6682/22591 | 80.1 | 0.000 | — | [35] |
| Chen M, 2015 | *NOS3*/rs1799983 | T vs. G | 1.05 (0.82-1.34) | — | Overall (Caucasian 3, Non-Caucasian 3) | Yes | 1055/877 | 64.4 | 0.015 | 0.707 | [35] |
| Chen M, 2015 | *NOS3*/rs1799983 | TT vs. GT+GG | 1.27 (0.96-1.68) | — | Overall (Caucasian 3, Non-Caucasian 3) | Yes | 1055/877 | 0 | 0.639 | — | [35] |
| Chen M, 2015 | *NOS3*/rs1799983 | TT+GT vs. GG | 1.03 (0.66-1.60) | — | Overall (Caucasian 3, Non-Caucasian 3) | Yes | 1055/877 | 80.8 | 0.000 | — | [35] |
| Chen M, 2015 | *NOS3*/rs1799983 | TT vs. GG | 1.29 (0.95-1.75) | — | Overall (Caucasian 3, Non-Caucasian 3) | Yes | 1055/877 | 6.9 | 0.372 | — | [35] |
| Chen M, 2015 | *NOS3*/rs1799983 | GT vs. GG | 0.97 (0.59-1.59) | — | Overall (Caucasian 3, Non-Caucasian 3) | Yes | 1055/877 | 82.8 | 0.000 | — | [35] |
| Chen H, 2015 | *DRD2*/rs1799732 | D vs. I | 1.05 (0.90-1.23) | — | Overall (Caucasian 1, Asian 1) | No | 535/664 | — | — | — | [34] |
| Chen H, 2015 | *DRD2*/rs1799732 | DD vs. DI + II | 11.35( 0.66-196.14) | — | Overall (Caucasian 1, Asian 1) | No | 535/664 | — | — | — | [34] |
| Chen H, 2015 | *DRD2*/rs1799732 | DD + DI vs. II | 1.18 (0.79-1.77) | — | Overall (Caucasian 1, Asian 1) | No | 535/664 | — | — | — | [34] |
| Chen H, 2015 | *DRD2*/rs1799732 | DD vs. II | 11.55 (0.67-199.78) | — | Overall (Caucasian 1, Asian 1) | No | 535/664 | — | — | — | [34] |
| Chen H, 2015 | *DRD2*/rs1799732 | DI vs. II | 1.09 (0.72-1.64) | — | Overall (Caucasian 1, Asian 1) | No | 535/664 | — | — | — | [34] |
| Chen H, 2015 | *DRD2*/rs6275 | G vs. A | 0.98 (0.84-1.15) | — | Overall (Caucasian 1, Asian 2) | No | 603/501 | — | — | — | [34] |
| Chen H, 2015 | *DRD2*/rs6275 | GG vs. AA + GA | 0.91 (0.67-1.24) | — | Overall (Caucasian 1, Asian 2) | No | 603/501 | — | — | — | [34] |
| Chen H, 2015 | *DRD2*/rs6275 | GG + GA vs. AA | 1.01 (0.83-1.22) | — | Overall (Caucasian 1, Asian 2) | No | 603/501 | — | — | — | [34] |
| Chen H, 2015 | *DRD2*/rs6275 | GG vs. AA | 0.94 (0.67-1.32) | — | Overall (Caucasian 1, Asian 2) | No | 603/501 | — | — | — | [34] |
| Chen H, 2015 | *DRD2*/rs6275 | GA vs. AA | 1.03 (0.82-1.28) | — | Overall (Caucasian 1, Asian 2) | No | 603/501 | — | — | — | [34] |
| Chen H, 2015 | *COMT*/rs4680 | A vs. G | 0.96 (0.85-1.09) | — | Overall (Caucasian 1, Asian 2) | No | 524/1626 | — | — | — | [34] |
| Chen H, 2015 | *COMT*/rs4680 | AA vs. AG + GG | 0.76 (0.60-0.97) | — | Overall (Caucasian 1, Asian 2) | No | 524/1626 | — | — | — | [34] |
| Chen H, 2015 | *COMT*/rs4680 | AA + AG vs. GG | 1.04 (0.89-1.21) | — | Overall (Caucasian 1, Asian 2) | No | 524/1626 | — | — | — | [34] |
| Chen H, 2015 | *COMT*/rs4680 | AA vs. GG | 0.97 (0.74-1.26) | — | Overall (Caucasian 1, Asian 2) | No | 524/1626 | — | — | — | [34] |
| Chen H, 2015 | *COMT*/rs4680 | AG vs. GG | 1.09 (0.91-1.31) | — | Overall (Caucasian 1, Asian 2) | No | 524/1626 | — | — | — | [34] |
| Chen H, 2015 | *DBH*/rs72393728 | D vs. I | 1.02 (0.93-1.12) | — | Overall (Caucasian 3, Asian 1) | No | 1382/1240 | — | — | — | [34] |
| Chen H, 2015 | *DBH*/rs72393728 | DD vs. DI + II | 0.98 (0.88-1.11) | — | Overall (Caucasian 3, Asian 1) | No | 1382/1240 | — | — | — | [34] |
| Chen H, 2015 | *DBH*/rs72393728 | DD + DI vs. II | 0.98 (0.88-1.11) | — | Overall (Caucasian 3, Asian 1) | No | 1382/1240 | — | — | — | [34] |
| Chen H, 2015 | *DBH*/rs72393728 | DD vs. II | 1.02 (0.86-1.22) | — | Overall (Caucasian 3, Asian 1) | No | 1382/1240 | — | — | — | [34] |
| Chen H, 2015 | *DBH*/rs72393728 | DI vs. II | 0.95 (0.82-1.10) | — | Overall (Caucasian 3, Asian 1) | No | 1382/1240 | — | — | — | [34] |
| Chen H, 2015 | *MAO-A*/VNTR | Allelic model | 0.97 (0.79-1.19) | — | Overall (Caucasian 2, Asian 1) | No | 242/279 | — | — | — | [34] |
| Chen H, 2015 | *MAO-A*/VNTR | Recessive model | 0.80 (0.57-1.13) | — | Overall (Caucasian 2, Asian 1) | No | 242/279 | — | — | — | [34] |
| Chen H, 2015 | *MAO-A*/VNTR | Dominant model | 1.06 (0.82-1.38) | — | Overall (Caucasian 2, Asian 1) | No | 242/279 | — | — | — | [34] |
| Chen H, 2015 | *MAO-A*/VNTR | Homozygous model | 1.01 (0.69-1.48) | — | Overall (Caucasian 2, Asian 1) | No | 242/279 | — | — | — | [34] |
| Chen H, 2015 | *MAO-A*/VNTR | Heterozygous model | 1.18 (0.84-1.66) | — | Overall (Caucasian 2, Asian 1) | No | 242/279 | — | — | — | [34] |
| Peng J, 2014 | *5HT2A*/T102C | T vs. C | 1.03 (0.87-1.22) | 0.74 | Overall (Caucasian 3, Asian 2) | Yes | 1156/1082 | 0 | 0.593 | 0.802 | [33] |
| Peng J, 2014 | *5HT2A*/T102C | TT vs. CC | 1.08 (0.76-1.54) | 0.66 | Overall (Caucasian 3, Asian 2) | Yes | 279/263 | 0 | 0.593 | 0.693 | [33] |
| Peng J, 2014 | *5HT2A*/T102C | TT + TC vs. CC | 1.07 (0.80-1.42) | 0.66 | Overall (Caucasian 3, Asian 2) | Yes | 578/541 | 0 | 0.941 | 0.490 | [33] |
| Peng J, 2014 | *5HT2A*/T102C | TT vs. TC + CC | 1.02 (0.77-1.34) | 0.90 | Overall (Caucasian 3, Asian 2) | Yes | 578/541 | 0 | 0.260 | 0.501 | [33] |
| Peng J, 2014 | *5HT2A*/A-1438G | A vs. G | 0.996 (0.73-1.37) | 0.98 | Overall (Caucasian 1, Asian 1) | Yes | 286/344 | 0 | 0.907 | — | [33] |
| Peng J, 2014 | *5HT2A*/A-1438G | AA vs. GG | 0.98 (0.51-1.89) | 0.96 | Overall (Caucasian 1, Asian 1) | Yes | 65/83 | 0 | 0.872 | — | [33] |
| Peng J, 2014 | *5HT2A*/A-1438G | AA + AG vs. GG | 1.12 (0.65-1.91) | 0.68 | Overall (Caucasian 1, Asian 1) | Yes | 143/172 | 0 | 0.823 | — | [33] |
| Peng J, 2014 | *5HT2A*/A-1438G | AA vs. AG + GG | 0.89 (0.53-1.50) | 0.66 | Overall (Caucasian 1, Asian 1) | Yes | 143/172 | 0 | 0.642 | — | [33] |
| Liu R, 2014 | *TNF-β*/rs909253 | G vs. A | 1.01 (0.97-1.06) | — | Overall (Caucasian 4, Asian 3) | Yes | 5557/20543 | — | 0.139 | — | [32] |
| Liu R, 2014 | *TNF-β*/rs909253 | GG vs. AG + AA | 1.01 (0.92-1.11) | — | Overall (Caucasian 4, Asian 3) | Yes | 5557/20543 | 35.1 | 0.160 | — | [32] |
| Liu R, 2014 | *TNF-β*/rs909253 | GG + AG vs. AA | 1.01 (0.96-1.06) | — | Overall (Caucasian 4, Asian 3) | Yes | 5557/20543 | — | 0.538 | — | [32] |
| Liu R, 2014 | *TNF-β*/rs909253 | GG vs. AA | 1.01 (0.92-1.12) | — | Overall (Caucasian 4, Asian 3) | Yes | 5557/20543 | — | 0.246 | 0.900 | [32] |
| Liu R, 2014 | *TNF-β*/rs909253 | AG vs. AA | 1.01 (0.96-1.07) | — | Overall (Caucasian 4, Asian 3) | Yes | 5557/20543 | — | 0.658 | — | [32] |
| Liu R, 2014 | *MTHFR*/rs1801133 | T vs. C | 1.00 (0.96-1.04) | — | Overall (Caucasian 12, Asian 4) | Yes | 8845/27496 | 22.2 | 0.201 | — | [31] |
| Liu R, 2014 | *MTHFR*/rs1801133 | TT vs. CT+CC | 1.11 (0.95-1.31) | — | Overall (Caucasian 12, Asian 4) | Yes | 8845/27496 | 49.2 | 0.014 | — | [31] |
| Liu R, 2014 | *MTHFR*/rs1801133 | TT+CT vs. CC | 1.00 (0.96-1.05) | — | Overall (Caucasian 12, Asian 4) | Yes | 8845/27496 | 0 | 0.917 | — | [31] |
| Liu R, 2014 | *MTHFR*/rs1801133 | TT vs. CC | 1.09 (0.93-1.29) | — | Overall (Caucasian 12, Asian 4) | Yes | 8845/27496 | 45.8 | 0.024 | 0.370 | [31] |
| Liu R, 2014 | *MTHFR*/rs1801133 | CT vs. CC | 1.01 (0.96-1.06) | — | Overall (Caucasian 12, Asian 4) | Yes | 8845/27496 | 0 | 0.945 | — | [31] |
| Tammimaki A, 2012 | *COMT*/rs4680 | — | 0.95 (0.81-1.11) | 0.52 | Overall (Caucasian 2, Asian 1) | No | 1237/1664 | 0 | 0.4 | 0.3 | [28] |
| Schurks M, 2011 | *TNF-α*/rs1800629 | AA vs. GG | 1.16 (0.80-1.68) | — | Overall (Caucasian 5, Asian 2, Heterogeneous 3) | Yes | 6360/22227 | 88.5 | <0.0001 | 0.68 | [25] |
| Schurks M, 2011 | *TNF-α*/rs1800629 | AA + AG vs. GG | 1.21 (0.81-1.80) | — | Overall (Caucasian 5, Asian 2, Heterogeneous 3) | Yes | 6360/22227 | 88.7 | <0.0001 | 0.65 | [25] |
| Schurks M, 2011 | *TNF-α*/rs1800629 | AA vs. GG+ AG | 0.95 (0.36-2.52) | — | Overall (Caucasian 5, Asian 2, Heterogeneous 3) | Yes | 6360/22227 | 59.9 | 0.03 | 0.99 | [25] |
| Schurks M, 2011 | *TNF-β*/rs909253 | GG vs. AA | 1.02 (0.87-1.21) | — | Overall (Caucasian 4, Asian 2) | Yes | 5466/20424 | 66 | 0.01 | 1.00 | [25] |
| Schurks M, 2011 | *TNF-β*/rs909253 | GG + GA vs. AA | 1.02 (0.84-1.23) | — | Overall (Caucasian 4, Asian 2) | Yes | 5466/20424 | 56.8 | 0.04 | 0.82 | [25] |
| Schurks M, 2011 | *TNF-β*/rs909253 | GG vs. AA+ GA | 1.06 (0.80-1.42) | — | Overall (Caucasian 4, Asian 2) | Yes | 5466/20424 | 46.6 | 0.10 | 0.89 | [25] |
| Samaan Z, 2011 | *MTHFR*/rs1801133 | TT vs. CC | 1.37 (1.07-1.76) | — | Overall (European 12, Non-European 3) | Yes | 4374/30110 | — | <0.0001 | 0.03 | [24] |
| Liu H,2011 | *5-HTTLPR* | S vs. L | 1.06 (0.95–1.17) | 0.31 | Overall (Caucasian 8, Asian 4) | Yes | 1502/1958 | 27 | 0.18 | — | [23] |
| Liu H,2011 | *5-HTTLPR* | SS + SL vs. LL | 1.04 (0.88–1.22) | 0.65 | Overall (Caucasian 8, Asian 4) | Yes | 1502/1958 | 23 | 0.22 | — | [23] |
| Liu H,2011 | *5-HTTLPR* | SS vs. SL + LL | 1.11 (0.94–1.32) | 0.23 | Overall (Caucasian 8, Asian 4) | Yes | 1502/1958 | 0 | 0.52 | 0.037 | [23] |
| Liu H,2011 | *5-HTT*/VNTR | 12 vs. other | 1.34 (1.09–1.64) | 0.006 | Overall (Caucasian 3, Asian 1) | Yes | 495/729 | 0 | 0.48 | — | [23] |
| Liu H,2011 | *5-HTT*/VNTR | 12/12 + 12/other vs. other | 1.27 (0.84–1.93) | 0.26 | Overall (Caucasian 3, Asian 1) | Yes | 495/729 | 41 | 0.17 | 0.864 | [23] |
| Liu H,2011 | *5-HTT*/VNTR | 12/12 vs. other + 12/other | 1.55 (1.17-2.05) | 0.002 | Overall (Caucasian 3, Asian 1) | Yes | 495/729 | 1 | 0.39 | — | [23] |
| Liu H,2011 | *5-HTT*/rs2020942 | T vs. C | 1.01 (0.87–1.16) | 0.92 | Caucasian 2 | No | 790/794 | 0 | 0.56 | — | [23] |
| Liu H,2011 | *5-HTT*/rs2020942 | TT + TC vs. CC | 1.09 (0.82–1.44) | 0.55 | Caucasian 2 | No | 790/794 | 0 | 0.91 | — | [23] |
| Liu H,2011 | *5-HTT*/rs2020942 | TT vs. CC+ TC | 0.97 (0.79–1.19) | 0.77 | Caucasian 2 | No | 790/794 | 0 | 0.38 | — | [23] |
| Schurks M, 2010 | *MTHFR*/rs180113 | TT vs. CC | 1.15 (1.00-1.31) | — | Overall (Caucasian 8, Turkish 1, Asian 1, Indian 1) | Yes | 6446/24578 | 66 | 0.001 | 0.03 | [20] |
| Schurks M, 2010 | *MTHFR*/rs180113 | TT + TC vs. CC | 1.08 (0.96-1.22) | — | Overall (Caucasian 8, Turkish 1, Asian 1, Indian 1) | Yes | 6446/24578 | 29 | 0.17 | 0.09 | [20] |
| Schurks M, 2010 | *MTHFR*/rs180113 | TT vs. CC+ TC | 1.39 (1.02-1.90) | — | Overall (Caucasian 8, Turkish 1, Asian 1, Indian 1) | Yes | 6446/24578 | 72 | <0.001 | 0.008 | [20] |
| Schurks M, 2010 | *ACE*/rs1799752 | II vs. DD | 0.93 (0.86-1.02) | — | Overall (Caucasian 3, Turkish 2, Asian 2, Indian 1) | Yes | 6120/22310 | 30 | 0.19 | 0.08 | [20] |
| Schurks M, 2010 | *ACE*/rs1799752 | II + ID vs. DD | 1.00 (0.94-1.07) | — | Overall (Caucasian 3, Turkish 2, Asian 2, Indian 1) | Yes | 6120/22310 | 0 | 0.70 | 0.12 | [20] |
| Schurks M, 2010 | *ACE*/rs1799752 | II vs. DD+ ID | 0.83 (0.69-1.01) | — | Overall (Caucasian 3, Turkish 2, Asian 2, Indian 1) | Yes | 6120/22310 | 58 | 0.02 | 0.12 | [20] |
| Schurks M, 2010 | *SLC6A4* STin2/VNTR | 10 vs. 12 | 1.00 (0.72–1.41) | — | Overall (European 3, Turkish 1) | Yes | 557/849 | 70.4 | 0.02 | 0.045 | [19] |
| Schurks M, 2010 | *SLC6A4 5*/HTTLPR | SS vs. LL | 1.16 (0.93–1.43) | — | European 6 | Yes | 651/1007 | 46.1 | 0.10 | 0.11 | [18] |
| Schurks M, 2010 | *SLC6A4* *5*/HTTLPR | SS vs. LL | 1.14 (0.85–1.55) | — | Asian 2 | Yes | 203/360 | 0 | 0.45 | — | [18] |
| Schurks M, 2010 | *SLC6A4* *5*/HTTLPR | SS + SL vs. LL | 1.16 (0.83–1.61) | — | European 6 | Yes | 651/1007 | 45.9 | 0.10 | 0.18 | [18] |
| Schurks M, 2010 | *SLC6A4* *5*/HTTLPR | SS + SL vs. LL | 1.30 (0.57–2.97) | — | Asian 2 | Yes | 203/360 | 0 | 0.92 | — | [18] |
| Schurks M, 2010 | *SLC6A4* *5*/HTTLPR | SS vs. LL+ SL | 1.27 (0.96–1.68) | — | European 6 | Yes | 651/1007 | 0 | 0.42 | 0.27 | [18] |
| Schurks M, 2010 | *SLC6A4* *5*/HTTLPR | SS vs. LL+ SL | 1.16 (0.81–1.66) | — | Asian 2 | Yes | 203/360 | 0 | 0.40 | — | [18] |
| Schurks M, 2010 | *ESR-1*/rs2228480 | AA vs. GG | 1.37 (1.02–1.83) | — | Caucasian 4 | Yes | 1061/926 | 71.6 | 0.01 | 0.44 | [17] |
| Schurks M, 2010 | *ESR-1*/rs2228480 | AA + AG vs. GG | 1.50 (1.10–2.06) | — | Caucasian 4 | Yes | 1061/926 | 64.5 | 0.04 | 0.47 | [17] |
| Schurks M, 2010 | *ESR-1*/rs2228480 | AA vs. GG+ AG | 1.34 (0.74–2.43) | — | Caucasian 4 | Yes | 1061/926 | 54.8 | 0.08 | 0.33 | [17] |
| Schurks M, 2010 | *ESR-1*/rs1801132 | GG vs. CC | 1.16 (1.03–1.32) | — | Overall (Caucasian 4, Indian 1) | Yes | 1381/1280 | 0 | 0.81 | 0.04 | [17] |
| Schurks M, 2010 | *ESR-1*/rs1801132 | GG + GC vs. CC | 1.16 (0.99–1.37) | — | Overall (Caucasian 4, Indian 1) | Yes | 1381/1280 | 0 | 0.75 | 0.30 | [17] |
| Schurks M, 2010 | *ESR-1*/rs1801132 | GG vs. CC+ GC | 1.40 (0.93–2.11) | — | Overall (Caucasian 4, Indian 1) | Yes | 1381/1280 | 38.9 | 0.16 | 0.80 | [17] |
| Schurks M, 2010 | *ESR-1*/rs2234693 | TT vs. CC | 1.31 (0.58–2.96) | — | Overall (Caucasian1, Indian 1) | Yes | 448/419 | 93.5 | <0.0001 | — | [17] |
| Schurks M, 2010 | *ESR-1*/rs2234693 | TT + TC vs. CC | 1.53 (0.60–3.92) | — | Overall (Caucasian1, Indian 1) | Yes | 448/419 | 89.4 | 0.002 | — | [17] |
| Schurks M, 2010 | *ESR-1*/rs2234693 | TT vs. CC+ TC | 1.20 (0.44–3.28) | — | Overall (Caucasian1, Indian 1) | Yes | 448/419 | 86.3 | 0.01 | — | [17] |
| Schurks M, 2010 | *PGR*/PROGINS insert | T2/T2 vs. T1/T1 | 1.02 (0.55–1.87) | — | Overall (Caucasian 3, Indian 1) | Yes | 936/906 | 87.5 | <0.0001 | 0.37 | [17] |
| Schurks M, 2010 | *PGR*/PROGINS insert | T2/T2 + T2/T1 vs. T1/T1 | 1.06 (0.53–2.09) | — | Overall (Caucasian 3, Indian 1) | Yes | 936/906 | 87.8 | <0.0001 | 0.35 | [17] |
| Schurks M, 2010 | *PGR*/PROGINS insert | T2/T2 vs. T1/T1 + T2/T1 | 1.22 (0.59–2.55) | — | Caucasian 3 | Yes | 936/906 | 0.7 | 0.37 | 0.56 | [17] |
| Rubino E, 2009 | *MTHFR*/C677T | T vs. C | 1.16 (0.97. 1.40) | — | Overall (Caucasian 5, Asian 1) | Yea | 2961/3844 | 65 | 0.01 | — | [15] |
| Rubino E, 2009 | *MTHFR*/C677T | TT vs. CC | 1.52 (0.96. 2.43) | — | Overall (Caucasian 5, Asian 1) | Yes | 2961/3844 | 69 | 0.006 | — | [15] |
| Rubino E, 2009 | *MTHFR*/C677T | TT vs. CT + CC | 1.40 (0.97. 2.02) | — | Overall (Caucasian 5, Asian 1) | Yes | 2961/3844 | 57 | 0.04 | — | [15] |
| Rubino E, 2009 | *MTHFR*/C677T | TT + CT vs. CC | 1.15 (0.92. 1.43) | — | Overall (Caucasian 5, Asian 1) | Yes | 2961/3844 | 54 | 0.06 | — | [15] |
| Oterino A, 2007 | *HTR2C*/Cys23Ser | Ser vs. Cys | 1.10 (0.76–1.58) | 0.498 | Overall (Caucasian 4, Asian 1) | No | 561/1235 | 46.14 | — | — | [14] |

OR: odds radio; CI: confidence interval;
