# Supplementary material for: Genetic variants in migraine: a field synopsis and systematic re-analysis of meta-analyses
Source: J Headache Pain. 2020 Feb 11;21(1):13. doi: 10.1186/s10194-020-01087-5 (PMC7011260; doi:10.1186/s10194-020-01087-5)
Supplement: Supplementary file 2 — Additional file 2: Table S2. Overall summary of results from meta-analyses of GWAS on risk of migraine (including duplicates, statistically significant and non-significant results). [file 10194_2020_1087_MOESM2_ESM.doc]

**Table S2. Overall summary of results from meta-analyses of GWAS on risk of migraine (including duplicates, statistically significant and non-significant results).**

| Author, Year | Gene/Variant | Comparison | OR (95%CI) | *P*-value | Ethnicity | No. of case/Control | *I*^2^ (%) | *I*^2^ (*P*) | Egger *P*-value | Reference |
| --- | --- | --- | --- | --- | --- | --- | --- | --- | --- | --- |
| Chang X, 2018 | *NMUR2*/rs1946225 | G vs. T | 2.29 (1.73-3.05) | 9.55E-09 | Overall (African-American, European-American) | 1212/13494 | — | — | — | [45] |
| Chang X, 2018 | *NMUR2*/rs72793414 | A vs. G | 2.44 (1.85-3.23) | 3.81E-10 | Overall (African-American, European-American) | 1212/13494 | — | — | — | [45] |
| Gormley P, 2016 | *LRP1*/rs11172113 | C vs. T | 0.90 (0.89-0.91) | 5.6E-49 | Caucasian 22 | 59674/316078 | — | — | — | [40] |
| Gormley P, 2016 | *PRDM16*/rs10218452 | G vs. A | 1.11 (1.10-1.13) | 5.3E-38 | Caucasian 22 | 59674/316078 | — | — | — | [40] |
| Gormley P, 2016 | *FHL5*/rs67338227 | T vs. A | 1.09 (1.08-1.11) | 2.0E-27 | Caucasian 22 | 59674/316078 | — | — | — | [40] |
| Gormley P, 2016 | *TSPAN2*/rs2078371 | C vs. T | 1.11 (1.09-1.13) | 4.1E-24 | Caucasian 22 | 59674/316078 | — | — | — | [40] |
| Gormley P, 2016 | *TRPM8*/rs10166942 | C vs. T | 0.94 (0.89-0.99) | 1.0E-23 | Caucasian 22 | 59674/316078 | — | — | — | [40] |
| Gormley P, 2016 | *PHACTR1*/rs9349379 | G vs. A | 0.93 (0.92-0.95) | 5.8E-22 | Caucasian 22 | 59674/316078 | — | — | — | [40] |
| Gormley P, 2016 | *MEF2D*/rs1925950 | A vs. G | 1.07 (1.06-1.09) | 9.1E-22 | Caucasian 22 | 59674/316078 | — | — | — | [40] |
| Gormley P, 2016 | *SLC24A3*/rs4814864 | C vs. G | 1.07 (1.06-1.09) | 2.2E-19 | Caucasian 22 | 59674/316078 | — | — | — | [40] |
| Gormley P, 2016 | *FGF6*/rs1024905 | A vs. G | 1.06 (1.04-1.08) | 2.1E-17 | Caucasian 22 | 59674/316078 | — | — | — | [40] |
| Gormley P, 2016 | *C7orf10*/rs186166891 | T vs. A | 1.09 (1.07-1.12) | 9.7E-16 | Caucasian 22 | 59674/316078 | — | — | — | [40] |
| Gormley P, 2016 | *PLCE1*/rs10786156 | G vs. C | 0.95 (0.94-0.96) | 2.0E-14 | Caucasian 22 | 59674/316078 | — | — | — | [40] |
| Gormley P, 2016 | *KCNK5*/rs10456100 | T vs. C | 1.06 (1.04-1.07) | 6.9E-13 | Caucasian 22 | 59674/316078 | — | — | — | [40] |
| Gormley P, 2016 | *ASTN2*/rs6478241 | T vs. A | 1.05 (1.04-1.07) | 1.2E-12 | Caucasian 22 | 59674/316078 | — | — | — | [40] |
| Gormley P, 2016 | *MRVI1*/rs4910165 | G vs. C | 0.94 (0.91-0.98) | 2.9E-11 | Caucasian 22 | 59674/316078 | — | — | — | [40] |
| Gormley P, 2016 | *HPSE2*/rs12260159 | A vs. G | 0.92 (0.89-0.94) | 3.2E-10 | Caucasian 22 | 59674/316078 | — | — | — | [40] |
| Gormley P, 2016 | *CFDP1*/rs77505915 | A vs. T | 1.05 (1.03-1.06) | 3.3E-10 | Caucasian 22 | 59674/316078 | — | — | — | [40] |
| Gormley P, 2016 | *RNF213*/rs17857135 | C vs. T | 1.06 (1.04-1.08) | 5.2E-10 | Caucasian 22 | 59674/316078 | — | — | — | [40] |
| Gormley P, 2016 | *NRP1*/rs2506142 | G vs. A | 1.06 (1.04-1.07) | 1.5E-09 | Caucasian 22 | 59674/316078 | — | — | — | [40] |
| Gormley P, 2016 | *GPR149*/rs13078967 | C vs. A | 0.87 (0.83-0.91) | 1.8E-09 | Caucasian 22 | 59674/316078 | — | — | — | [40] |
| Gormley P, 2016 | *JAG1*/rs111404218 | G vs. C | 1.05 (1.03-1.07) | 2.0E-09 | Caucasian 22 | 59674/316078 | — | — | — | [40] |
| Gormley P, 2016 | *SPINK2*/rs7684253 | T vs. C | 0.96 (0.94-0.97) | 2.5E-09 | Caucasian 22 | 59674/316078 | — | — | — | [40] |
| Gormley P, 2016 | *ZCCHC14*/rs4081947 | G vs. A | 1.03 (1.00-1.06) | 2.5E-09 | Caucasian 22 | 59674/316078 | — | — | — | [40] |
| Gormley P, 2016 | *HEY2*/rs1268083 | C vs. T | 0.96 (0.95-0.97) | 5.3E-09 | Caucasian 22 | 59674/316078 | — | — | — | [40] |
| Gormley P, 2016 | *WSCD1*/rs75213074 | T vs. C | 0.89 (0.86-0.93) | 7.1E-09 | Caucasian 22 | 59674/316078 | — | — | — | [40] |
| Gormley P, 2016 | *GJA1*/rs28455731 | T vs. G | 1.06 (1.04-1.08) | 7.3E-09 | Caucasian 22 | 59674/316078 | — | — | — | [40] |
| Gormley P, 2016 | *TGFBR2*/rs6791480 | T vs. C | 1.04 (1.03-1.06) | 7.8E-09 | Caucasian 22 | 59674/316078 | — | — | — | [40] |
| Gormley P, 2016 | *ITPK1*/rs11624776 | C vs. A | 0.96 (0.94-0.97) | 7.9E-09 | Caucasian 22 | 59674/316078 | — | — | — | [40] |
| Gormley P, 2016 | *ADAMTSL4*/rs6693567 | T vs. C | 1.05 (1.03-1.06) | 1.2E-08 | Caucasian 22 | 59674/316078 | — | — | — | [40] |
| Gormley P, 2016 | *CCM2L*/rs144017103 | T vs. C | 0.85 (0.76-0.96) | 1.2E-08 | Caucasian 22 | 59674/316078 | — | — | — | [40] |
| Gormley P, 2016 | *YAP1*/rs10895275 | A vs. T | 1.04 (1.03-1.06) | 1.6E-08 | Caucasian 22 | 59674/316078 | — | — | — | [40] |
| Gormley P, 2016 | *MED14*/rs12845494 | G vs. C | 0.96 (0.95-0.97) | 1.7E-08 | Caucasian 22 | 59674/316078 | — | — | — | [40] |
| Gormley P, 2016 | *DOCK4*/rs10155855 | T vs. A | 1.08 (1.05-1.12) | 2.1E-08 | Caucasian 22 | 59674/316078 | — | — | — | [40] |
| Gormley P, 2016 | *LRRIQ3*/rs1572668 | G vs. A | 1.04 (1.02-1.05) | 2.1E-08 | Caucasian 22 | 59674/316078 | — | — | — | [40] |
| Gormley P, 2016 | *CARF*/rs138556413 | G vs. A | 0.88 (0.84-0.92) | 2.3E-08 | Caucasian 22 | 59674/316078 | — | — | — | [40] |
| Gormley P, 2016 | *ARMS2*/rs2223089 | C vs. G | 0.93 (0.91-0.95) | 3.0E-08 | Caucasian 22 | 59674/316078 | — | — | — | [40] |
| Gormley P, 2016 | *IGSF9B*/rs561561 | T vs. A | 0.94 (0.92-0.96) | 3.4E-08 | Caucasian 22 | 59674/316078 | — | — | — | [40] |
| Gormley P, 2016 | *MPPED2*/rs11031122 | C vs. T | 1.04 (1.03-1.06) | 3.5E-08 | Caucasian 22 | 59674/316078 | — | — | — | [40] |
| Gormley P, 2016 | *NOTCH4*/rs140002913 | A vs. G | 0.91 (0.88-0.94) | 3.8E-08 | Caucasian 22 | 59674/316078 | — | — | — | [40] |
| Esserlind AL, 2013 | *TSPAN-2*/rs2078371 | C vs. T | 1.14 (1.09-1.20) | 2.55E-08 | Caucasian 6 | 11473/70227 | — | — | — | [30] |
| Esserlind AL, 2013 | *PRDM16*/rs2651899 | C vs. T | 1.10 (1.06-1.13) | 9.27E-09 | Caucasian 6 | 11473/70227 | — | — | — | [30] |
| Esserlind AL, 2013 | *TRPM8*/rs10166942 | C vs. T | 0.86 (0.82-0.89) | 1.03E-13 | Caucasian 6 | 11473/70227 | — | — | — | [30] |
| Esserlind AL, 2013 | *LRP1*/rs11172113 | T vs. C | 0.90 (0.87-0.93) | 2.06E-11 | Caucasian 6 | 11473/70227 | — | — | — | [30] |
| Anttila V, 2013 | *PRDM16*/rs2651899 | C vs. T | 1.09 (1.07-1.12) | 3.28E-14 | Caucasian 19 | 23285/95425 | 20 | 0.214 | — | [29] |
| Anttila V, 2013 | near *AJAP1*/rs10915437 | G vs. A | 0.86 (0.82-0.91) | 2.81E-08 | Caucasian 5 | 5175/13 972 | 47 | 0.108 | — | [29] |
| Anttila V, 2013 | near *TSPAN2*/rs12134493 | A vs. C | 1.14 (1.10-1.18) | 6.71E-14 | Caucasian 19 | 23285/95425 | 14 | 0.408 | — | [29] |
| Anttila V, 2013 | *MEF2D*/rs2274316 | A vs. C | 1.07 (1.04-1.09) | 3.14E-08 | Caucasian 19 | 23285/95425 | 45 | 0.021 | — | [29] |
| Anttila V, 2013 | *TRPM8*/rs7577262 | A vs. G | 0.87 (0.84-0.90) | 3.27E-13 | Caucasian 19 | 23285/95425 | 33 | 0.070 | — | [29] |
| Anttila V, 2013 | near *TGFBR2*/rs6790925 | T vs. C | 1.15 (1.10-1.21) | 2.16E-08 | Caucasian 5 | 5175/13 972 | 0 | 0.780 | — | [29] |
| Anttila V, 2013 | *PHACTR1*/rs9349379 | G vs. A | 0.86 (0.82-0.90) | 2.81E-10 | Caucasian 11 | 7107/69427 | 0 | 0.443 | — | [29] |
| Anttila V, 2013 | *FHL5*/rs13208321 | G vs. A | 1.18 (1.13-1.24) | 2.15E-12 | Caucasian 19 | 23285/95425 | 0 | 0.168 | — | [29] |
| Anttila V, 2013 | *c7orf10*/rs4379368 | T vs. C | 1.11 (1.08-1.15) | 1.46E-09 | Caucasian 19 | 23285/95425 | 2 | 0.441 | — | [29] |
| Anttila V, 2013 | near *MMP16*/rs10504861 | T vs. C | 0.86 (0.81-0.90) | 1.32E-08 | Caucasian 11 | 7107/69427 | 0 | 0.755 | — | [29] |
| Anttila V, 2013 | *ASTN2*/rs6478241 | G vs. A | 1.16 (1.11-1.22) | 1.04E-09 | Caucasian 5 | 5175/13 972 | 0 | 0.646 | — | [29] |
| Anttila V, 2013 | *LRP1*/rs11172113 | C vs. T | 0.90 (0.88-0.92) | 2.69E-19 | Caucasian 19 | 23285/95425 | 22 | 0.188 | — | [29] |
| Ligthart L, 2011 | *NGFR*/rs9908234 | A vs. G | — | 8.00E-08 | Caucasian 6 | 2446/8534 | — | — | — | [22] |
| Ligthart L, 2011 | *AGBL1*/rs11636768 | A vs. G | — | 3.23E-07 | Caucasian 6 | 2446/8534 | — | — | — | [22] |
| Ligthart L, 2011 | *MACC1*/rs10275320 | A vs. G | — | 1.56E-06 | Caucasian 6 | 2446/8534 | — | — | — | [22] |
| Ligthart L, 2011 | *LIPG*/rs4939879 | A vs. G | — | 1.82E-06 | Caucasian 6 | 2446/8534 | — | — | — | [22] |
| Ligthart L, 2011 | *AGA*/rs4861775 | A vs. C | — | 3.28E-06 | Caucasian 6 | 2446/8534 | — | — | — | [22] |
| Ligthart L, 2011 | *KIF20B*/rs986222 | A vs. G | — | 3.37E-06 | Caucasian 6 | 2446/8534 | — | — | — | [22] |
| Ligthart L, 2011 | *BMP2*/rs6107848 | A vs. G | — | 5.90E-06 | Caucasian 6 | 2446/8534 | — | — | — | [22] |
| Ligthart L, 2011 | *IGLL1*/rs140174 | A vs. G | — | 6.98E-06 | Caucasian 6 | 2446/8534 | — | — | — | [22] |
| Ligthart L, 2011 | *TSPAN2*/rs1146161 | A vs. C | — | 9.27E-06 | Caucasian 6 | 2446/8534 | — | — | — | [22] |
| Ligthart L, 2011 | *KDM4C*/rs4742323 | C vs. G | — | 9.70E-06 | Caucasian 6 | 2446/8534 | — | — | — | [22] |
| Chasman DI, 2011 | *PRDM16*/rs2651899 | A vs. T | 1.11 (1.07–1.15) | 3.8E-09 | European 4 | 8950/32057 | 18.9 | 0.30 | — | [21] |
| Chasman DI, 2011 | —/ rs2078371 | C vs. T | 1.14 (1.08–1.20) | 6.9E-07 | European 4 | 8950/32057 | 0 | 0.53 | — | [21] |
| Chasman DI, 2011 | *TRPM8*/rs10166942 | C vs. T | 0.85 (0.82–0.89) | 5.5E-12 | European 4 | 8950/32057 | 0 | 0.90 | — | [21] |
| Chasman DI, 2011 | *SEPT7*/rs17172526 | A vs. G | 1.18 (1.10–1.26) | 1.4E-06 | European 4 | 8950/32057 | 0 | 0.67 | — | [21] |
| Chasman DI, 2011 | *C8orf79*/rs2203834 | C vs. A | 0.89 (0.85–0.94) | 8.1E-06 | European 4 | 8950/32057 | 67.7 | 0.03 | — | [21] |
| Chasman DI, 2011 | —/rs13290757 | T vs. C | 0.91 (0.86–0.96) | 2.6E-04 | European 4 | 8950/32057 | 72.1 | 0.01 | — | [21] |
| Chasman DI, 2011 | *LRP1*/rs11172113 | C vs. T | 0.90 (0.87–0.93) | 4.3E-09 | European 4 | 8950/32057 | 0 | 0.57 | — | [21] |
| Anttila, V. 2010 | *MTDH*/rs1835740 | C vs. T | 1.18 (1.13-1.24) | 1.60E-11 | Caucasian 7 | 5950/50809 | — | — | — | [16] |

GWAS: genome-wide association studies; OR: odds radio; CI: confidence interval
